# Entrepreneurs of conflict: A descriptive analysis of when and how political elites use divisive rhetoric

**DOI:** 10.1093/pnasnexus/pgag038

**Published:** 2026-03-17

**Authors:** Marc S Jacob, Yphtach Lelkes, Sean J Westwood

**Affiliations:** Keough School of Global Affairs, University of Notre Dame, 1010 Jenkins Nanovic Halls, Notre Dame, IN 46556, USA; Annenberg School for Communication, University of Pennsylvania, 3620 Walnut Street, Philadelphia, PA 19104, USA; Department of Government, Dartmouth College, Silsby Hall (3 Tuck Mall), Hanover, NH 03755, USA

## Abstract

The rise of divisive rhetoric in American politics reflects and reinforces broader system-level polarization, yet the incentives driving this behavior are not well understood. To investigate this puzzle, we conduct a large-scale descriptive analysis, linking 2.2 million public statements from the 118th US Congress to records of media coverage, campaign finance, and electoral outcomes. Using a large-language model, we identify “conflict entrepreneurs”—legislators who frequently use personal insults—and document their behavior and its correlates. Our descriptive findings reveal a stark but asymmetric pattern: personal attacks occur in both parties but are more frequent among Republicans and are strongly associated with greater media coverage but show no corresponding positive relationship with fundraising, vote margins, legislative success, or personal wealth. Furthermore, this rhetorical style does not simply reflect constituent sentiment; we find no correlation between a legislator’s use of insults and the partisan animosity in their district. These documented patterns suggest a political incentive structure where the pursuit of media visibility alone sustains a form of discourse that may be corrosive to democratic norms, even without apparent electoral or financial rewards.

Significance Statement Political polarization threatens democratic stability, yet the incentives for elite incivility are poorly understood. This study analyzes over 2 million statements from US legislators, using a large-language model to distinguish personal attacks from policy debate. We show that “conflict entrepreneurs”—politicians who specialize in personal insults—reap significant media attention but gain no measurable advantage in elections, fundraising, or lawmaking. Their rhetoric is also disconnected from their constituents’ attitudes. This reveals that media incentives, rather than electoral pressures, can promote toxic discourse. This dynamic, where visibility is decoupled from political accountability, has broad implications for understanding how communication ecosystems can degrade democratic institutions.

“The most recent additions to Congress don’t care about policy; they care about getting attention.”—Retired member of Congress

## Introduction

American voters consistently express aversion to political incivility.Survey after survey reveals that citizens want legislators to engage in substantive policy debate rather than personal attacks, preferring compromise to conflict and civility to confrontation. Yet, despite this clear public preference, vitriolic rhetoric appears to be increasing in American politics—legislators are engaging in name-calling, character assassination, and ad hominem attacks at seemingly unprecedented rates. This disconnect between citizen demand for civility and elite supply of conflict presents a fundamental puzzle for democratic theory: Why do political elites engage in behavior their constituents claim to despise?

Criticism of political opponents’ records is a long-standing feature of democratic representation, widely viewed as a means for voters to assess competing visions of public policy (1). In recent years, however, public discourse has shifted from issue-based debate toward personalized insults and defamation (2–5), prompting concerns about the health of deliberative norms and the resilience of political institutions (2–8). The shift toward more hostile rhetoric, in turn, is even more concerning given its range of adverse implications for democratic societies, including increased partisan animosity, support for political violence (9–11), and reduced political participation (12, pp. 203–228).

This trend is often explained by a set of common assumptions. Scholarly and popular commentary assumes that personal attacks occupy a large share of legislators’ communications, magnified by editorial selection and algorithmic amplification (13, 14). Many officeholders, in turn, appear convinced that such rhetoric yields tangible rewards in fundraising and electoral prospects, despite mixed empirical evidence and voter disapproval (12, 15–21). Other accounts suggest that legislators skillfully blend policy advocacy with partisan attacks or that negativity is primarily a feature of election seasons (12, 21, 22).

In this article, we systematically test these assumptions through a large-scale descriptive analysis of elite communication. We construct a novel dataset linking over two million public statements from the 118th Congress—including floor speeches, social media posts, newsletters, and press releases—to comprehensive records of members’ political, legislative, and financial activities. Using a large-language model (LLM), we develop a typology that distinguishes traditional *policy entrepreneurs* from *conflict entrepreneurs*—the latter defined as representatives who disproportionately use personal insults against the integrity, morality, or intellect of their peers. For instance, conflict entrepreneurs make statements like “Joe Biden is Hitler”^a^ or “Your boss [Trump] failed Pictionary when he couldn’t tell the difference between his ex-wife and a woman he assaulted in a dressing room. THE END!”^b^

Our findings reveal a critical insight about the heterogeneous objectives of modern legislators. While traditional theories of legislative behavior assume members primarily seek reelection, policy influence, or institutional power within Congress, we document a subset of legislators who appear to optimize for a different goal entirely: media visibility. This pursuit of visibility, however, appears decoupled from traditional political rewards. We find that while conflict entrepreneurs gain substantial media attention, this visibility is not associated with measurable gains in fundraising, electoral margins, legislative productivity, or personal wealth. Furthermore, their rhetorical style shows no correlation with the baseline partisan hostility of their constituents. These descriptive findings challenge the conventional wisdom about the benefits of political vitriol, pointing to an incentive structure where the pursuit of media visibility is decoupled from traditional electoral and financial rewards.

To frame our descriptive analysis of these assumptions, we integrate three complementary theoretical perspectives into a single narrative. First, media scholarship explains why personal attacks command outsized attention: commercial news producers and journalistic norms favor conflictual and sensational language, and psychological research on negativity bias shows that audiences focus disproportionately on hostile content (23, 24). Second, political entrepreneurship theory clarifies that legislators face distinct strategic roles: *policy entrepreneurs* devote rhetorical capital to substantive proposals and exploit policy windows to advance institutional innovations (25, 26). We expand this model to suggest that *conflict entrepreneurs* expend effort on personal denigration to capture media and public attention. Finally, social identity and affective polarization frameworks suggest that personal attacks trigger in-group solidarity and out-group hostility, intensifying partisan affect and undermining cross-party deliberation (27, 28). Together, these perspectives imply that conflict rhetoric is both highly visible and socially corrosive, yet may yield few substantive policy or electoral returns.

The article unfolds in four parts. First, we review existing theories of representational style—spanning media logic, political entrepreneurship, and deliberative-democracy norms—and articulate our descriptive expectations. Second, we describe our data collection procedures, LLM-based classification methods, and the construction of our conflict entrepreneur typology. Third, we present the distribution and correlates of conflict entrepreneurship across legislators. Finally, we discuss the broader implications of our findings for media practices, party strategies, and the durability of democratic norms.

## Theoretical framework

Political scientists have long recognized that journalistic norms shape the visibility of political language. News organizations, operating under commercial pressures, tend to select for statements that emphasize conflict and sensationalism (23, 29). Audiences, meanwhile, exhibit a well-documented *negativity bias*, attending more closely to hostile content than to neutral policy discussion (24, 30). Together, media logic and audience psychology create an environment where personal attacks are disproportionately highlighted, regardless of their actual frequency.

A second perspective, from the literature on political entrepreneurship, distinguishes actors who invest in substantive policy from those who deploy controversy as a strategic resource (31). Mintrom (26) describe *policy entrepreneurs* as individuals who leverage windows of opportunity to advance innovative ideas, building reputations on expertise (25). By contrast, we conceptualize *conflict entrepreneurs* as those who concentrate their rhetorical capital on personal attacks, treating derogatory messaging as a currency for attracting media attention and signaling partisan loyalty (32). From the media’s perspective, which often portrays political competition and discourse more as a game between opponents rather than a substantive process, incivility among politicians provides useful material for news reporting, thereby creating an additional incentive for politicians to engage in such behavior (33, 34). Overall, whereas policy entrepreneurship is theorized to correlate with legislative success, conflict entrepreneurship is expected to align more closely with media coverage and symbolic partisanship, without corresponding gains in policy influence.

Finally, theories of affective polarization and social identity help explain why such rhetoric may persist even if it yields few tangible policy returns. In highly polarized environments, personal attacks can function as powerful signals of in-group solidarity and out-group hostility, reinforcing partisan identities and mobilizing core supporters (27, 28). Beyond simple expressions of hostility, uncivil rhetoric can communicate clear boundaries between in-groups (e.g. members of the politician’s party) and out-groups (e.g. members of the other party), thereby creating and sustaining sharp perceptual differences between the groups. Politicians may thus strategically invoke identity cues to strengthen affective attachments among copartisans by stigmatizing opponents as outsiders through unambiguous uncivil attacks (31, 35). Conflict entrepreneurship is thus not only about sparking attention but also about activating shared identities that consolidate partisan solidarity by speaking derogatorily about rival groups. Legislators in electorally competitive or ideologically sorted districts may therefore perceive an advantage to using hostile language, regardless of its effect on cross-party deliberation or policy outcomes (36, 37).

These three perspectives generate a set of descriptive expectations that guide our analysis. First, we expect that representatives who rely on conflictual rhetoric will receive disproportionately high levels of media coverage relative to their policy-focused counterparts (23, 30). Second, this elevated visibility will not necessarily coincide with superior legislative sponsorship, fundraising totals, or committee assignments (26, 32). Third, we expect the prevalence of conflict entrepreneurship to be greatest among legislators from highly polarized constituencies, where signaling partisan loyalty carries particular weight (36, 37).

## Personal attacks and representation

Some legislators appear to view personal attacks on opponents as a means of increasing visibility and appealing to partisan audiences (38–41). As negative rhetoric in Congress has grown more frequent, scholars debate its effect on public attitudes and the quality of elite deliberation. On one hand, some evidence suggests no significant association between personal insults and citizen trust or political efficacy (16, 42). On the other, exposure to derogatory language has been linked to lower evaluations of substantive debate and higher levels of political cynicism (43, 44). Experimental studies further show that voters recall negative appeals more readily than positive messages, highlighting the particular salience of hostile communication (1, 45).

To trace the origins of these debates, early research focused on campaign-period negativity, examining how critiques of an opponent’s record, background, or character were associated with turnout and vote choice (46, 47). This body of work established that while harsh rhetoric could mobilize partisans, it also risked alienating undecided voters. Building on these foundations, recent studies have documented a clear extension of personal attacks into legislative and governing contexts (2, 3). This marks a departure from the mid-20th-century norms of policy-focused debate in Congress, a shift illustrated by incidents such as Rep. Joe Wilson (R-SC) shouting, “You lie!” at President Barack Obama, or Rep. Marjorie Taylor Greene (R-GA) telling Rep. Jasmine Crockett (D-TX), “I think your fake eyelashes are messing up what you’re reading.”

More broadly, today’s conflictual rhetoric appears across a range of venues—including floor speeches (2, 3), press releases, social media posts (38, 48), and televised interviews (49), suggesting a transformation in representational style that transcends electoral cycles. This expansion of negativity beyond campaigns underscores the need for a systematic, descriptive analysis of when and how personal attacks are employed and how they correspond to institutional behaviors and audience responses in the contemporary Congress.

The persistence of vitriolic rhetoric outside traditional campaign periods suggests that American politics has entered what Sydney (50) termed the “permanent campaign”—a state where the distinction between governing and campaigning has largely dissolved. Rather than viewing nonelection-period negativity as anomalous, our findings align with scholarship documenting how modern legislators operate in perpetual campaign mode, where electoral proximity has become a less meaningful predictor of political behavior (51). This permanent campaign framework helps explain why personal attacks remain constant features of congressional discourse regardless of electoral calendars, as legislators continuously cultivate media attention and partisan audiences rather than cycling between distinct governing and campaigning phases.

## Attention over substance

The literature suggests that politicians often adopt aggressive rhetoric based on the perception that negativity boosts their visibility and distinguishes their party’s platform (18, 52). Yet systematic studies find limited associations between such messaging and electoral gains (20), and surveys consistently report widespread voter disapproval of personal insults in politics (17).

This raises a puzzle: why does the behavior persist if it does not yield votes? The leading explanation is that the primary reward is not electoral but attentional. Observational evidence indicates that negative language correlates with heightened media coverage and intensified engagement among core supporters (12, 21). For example, content analyses show that messages framed in conflictual terms are more likely to be selected and amplified by journalists (53). Similarly, analyses of presidential communication show that spikes in attention to certain tweets coincide with subsequent shifts in news coverage—a pattern sometimes described as agenda diversion (54). In this context, a reliance on personal attacks may be a rational strategy for gaining visibility in a crowded media environment.

However, direct tests of this attention hypothesis are limited. Prior research has often relied on sentiment or incivility measures that do not distinguish between strong policy opposition and personal attacks. Moreover, the hypothesis has been tested primarily using social media engagement, particularly on X (formerly Twitter) (12, 55), rather than in other impactful media like cable news. Consequently, the key prediction—that such rhetoric garners media attention without incurring electoral punishment—remains largely untested with comprehensive data.

## A new approach to measuring what legislators say

Not all negativity is harmful to political discourse. From both normative and empirical perspectives, criticizing policy differences—including an opponent’s proposals or record—can inform voters and is a legitimate part of democratic debate (56, 57). Personal insults, however, are distinct; they undermine the quality of debate and likely exacerbate partisan animosity among both political elites and the public.

Capturing this distinction computationally has been a persistent challenge. Previous research focusing on the overall valence of political speech, using widely used sentiment classifiers like Vader or TextBlob (58, 59), often cannot separate policy-related negativity (i.e. objecting to the merits of a policy with negative language) from personal insults. An alternative approach, flagging all mentions of the opposing party, is imprecise, as the majority of such references are procedural rather than derogatory.

### Personal attack classification methodology

Our approach addresses these limitations using a LLM to classify political speech with high contextual accuracy. We constructed a large text corpus of public statements from every member of the 118th Congress, collecting daily data from floor speeches, press releases, newsletters, and posts on X (formerly Twitter). We segmented this text into roughly two-sentence units and used GPT-4o to classify each segment.^c^

We classify personal attacks using a LLM with the following operational definition: A personal attack is any statement that (i) targets a specific individual or group by name or clear reference and (ii) criticizes personal characteristics, motivations, or integrity rather than policy positions or actions in official capacity.

Our classification prompt specifically instructs the model to identify statements containing: (i) questioning of character, honesty, or patriotism; (ii) derogatory nicknames or personal insults; (iii) attacks on appearance, background, or personal life; (iv) accusations of corruption or malicious intent without policy context; and (v) inflammatory rhetoric targeting individuals rather than ideas.

Conversely, the following are explicitly **not** classified as personal attacks: (i) criticism of policy positions or votes; (ii) Disagreement with official actions or statements; (iii) factual descriptions of political affiliations or records; and (iv) general partisan criticism without individual targeting.

#### Methodological neutrality and factual claims

Our classification of personal attacks maintains methodological neutrality regarding the factual accuracy of accusations. When a legislator accuses another of being “antidemocratic,” “corrupt,” or “criminal,” we classify this as a personal attack regardless of whether evidence supports such claims. This approach recognizes that democratic discourse benefits when legislators critique specific policies, votes, or actions rather than impugning character or motives—even when concerns about antidemocratic behavior or criminal conduct may be legitimate.

For instance, a legislator concerned about colleagues’ actions on 2021 January 6, could constructively state: “Senator X’s vote to reject certified election results undermined democratic institutions” rather than “Senator X is antidemocratic.” Similarly, concerns about corruption are better expressed as “Representative Y accepted illegal campaign contributions” rather than “Representative Y lacks integrity.” The former approaches foster accountability through specific, falsifiable claims; the latter resort to character assassination that, regardless of merit, degrades discourse and may be strategically deployed even when factually questionable.

Our classification also encompasses attacks on political groups when they target members’ character rather than policies. Statements like “Republicans are fascists” or “Democrats are communists” constitute personal attacks against group members, as they impugn the character and motivations of individuals based on party affiliation rather than engaging with specific policy positions. While group-based attacks may seem less personal than individual targeting, they contribute to the same degradation of discourse by substituting character assassination for substantive debate.

We acknowledge this creates analytical tensions: some legislators have been convicted of crimes or sanctioned for ethics violations, making certain character-based criticisms factually grounded. However, our interest lies not in adjudicating truth claims but in understanding how legislators choose between policy-focused and person-focused rhetoric. Even when targeting genuinely problematic behavior, the choice to attack the person rather than the action represents a rhetorical strategy that prioritizes conflict over constructive democratic debate.

### Data sources

X/Twitter data were collected through the official application programming interface (API). Floor speeches were collected from the Library of Congress and processed with custom scripting. Newsletters came from the DCInbox tool. Press releases were collected and processed using custom scripting. The TV transcript data were provided by the Internet Archive through granted use of their raw API. All data collection was exhaustive (covering the full period of the 118th Congress).

### Model selection and testing

We employ GPT-4o for classification after systematic comparison of available LLMs. Our classification process began with earlier OpenAI models when competitive alternatives were not yet available. Upon the release of GPT-4o, we reclassified our data and found it significantly outperformed alternatives. We evaluated Llama and Claude models, but neither matched GPT-4o’s performance at the time of testing. While model capabilities continue to evolve, we maintain consistency with GPT-4o to avoid midstream changes and associated computational costs.

#### Human validation procedure

Our validation employed two human coders, both holding PhDs in political science. The coders worked independently using the same classification prompt provided to the GPT-4o model, ensuring direct comparability between human and machine classification (see Tables S1 and S2).

The validation process proceeded as follows:

1. Both coders independently classified a random sample of 500 statements from our corpus, using the same prompt as the LLM.
2. Where coders disagreed (initial agreement rate: 92% for personal attacks and 80% for critical debate), they resolved discrepancies through discussion to establish ground truth.
3. This consensus coding served as the gold standard for evaluating model performance.
4. For personal attacks model performance was: 97% accuracy, 98% precision, and 92% recall. For critical debate, the model achieved accuracy of 81%, precision of 84%, and recall of 92%.

### Limitations

Our classification approach faces several important limitations that warrant careful consideration. First, boundary cases where criticism blends personal and policy elements pose inherent challenges for any coding scheme. A statement like “Senator X’s reckless economic proposals demonstrate poor judgment” contains both policy critique and character assessment, making clean categorization difficult. The interpretation of sarcasm or implied criticism is highly context-dependent, and our model may inconsistently classify statements where tone or implicit meaning diverges from literal content. Additionally, distinguishing legitimate character questions relevant to fitness for office from pure personal attacks presents both methodological and normative challenges. Questioning a legislator’s integrity following criminal charges addresses relevant qualifications for office, yet may be coded identically to baseless character assassination, as our approach prioritizes rhetorical form over factual content.

Our text-based classification approach has important scope limitations that likely lead to systematic undercounting of personal attacks. We cannot capture implicit attacks and insinuations that denigrate individuals through implication rather than explicit naming, such as social media posts questioning the circumstances of the attack on Nancy Pelosi’s husband through innuendo rather than direct statements about Speaker Pelosi. Coded language and dog whistles pose similar challenges, as attacks embedded in seemingly neutral language may carry derogatory meaning to specific audiences while appearing innocuous in literal transcription. Visual and multimedia content including memes, manipulated images, and video presentations that may contain personal attacks fall entirely outside our text-only approach. Context-dependent sarcasm requires broader conversational or cultural context to identify as attacks, which our statement-level analysis cannot reliably capture. Finally, indirect targeting through attacks on family members, staff, or associates as proxies for the legislator themselves represents a form of personal rhetoric that our operationalization does not systematically identify.

Our classification scheme also does not systematically capture attacks based on physical appearance, body characteristics, race, ethnicity, or other identity-based attributes unless they constitute clear ad hominem rhetoric. This represents a critical direction for future research, as these forms of attacks may constitute a particularly pernicious form of political incivility that undermines democratic representation and may be disproportionately directed at women and minority legislators. These limitations mean our estimates should be interpreted as a lower bound on the true prevalence of personal attacks in congressional discourse, capturing only the most explicit and unambiguous instances of ad hominem rhetoric.

Our use of commercial LLMs for text classification introduces additional methodological considerations. The black-box nature of commercial LLMs means the exact decision-making process underlying each classification remains opaque, limiting our ability to fully understand or audit classification decisions (60, 61). Replicability presents a related challenge, as commercial LLM providers regularly update their models, meaning future replication attempts may obtain different results even with identical prompts (62). To address these concerns, we document specific model versions used, provide exact prompts in Supplementary materials, and archive all classification outputs to ensure the specific coding used in our analysis remains accessible. While LLMs demonstrate strong performance on many text classification tasks, they may exhibit biases or inconsistencies that differ from human coders (63).

The proprietary nature of commercial LLMs raises questions about scientific transparency that open-source alternatives may eventually address, though current open-source models lag in performance on complex classification tasks. For complete classification details, including full prompt text and validation results, see SI Section 2. For examples, see SI Table S3 and Table S4.

### Comparison of our measure against standard classifiers

**Fig. 1.**
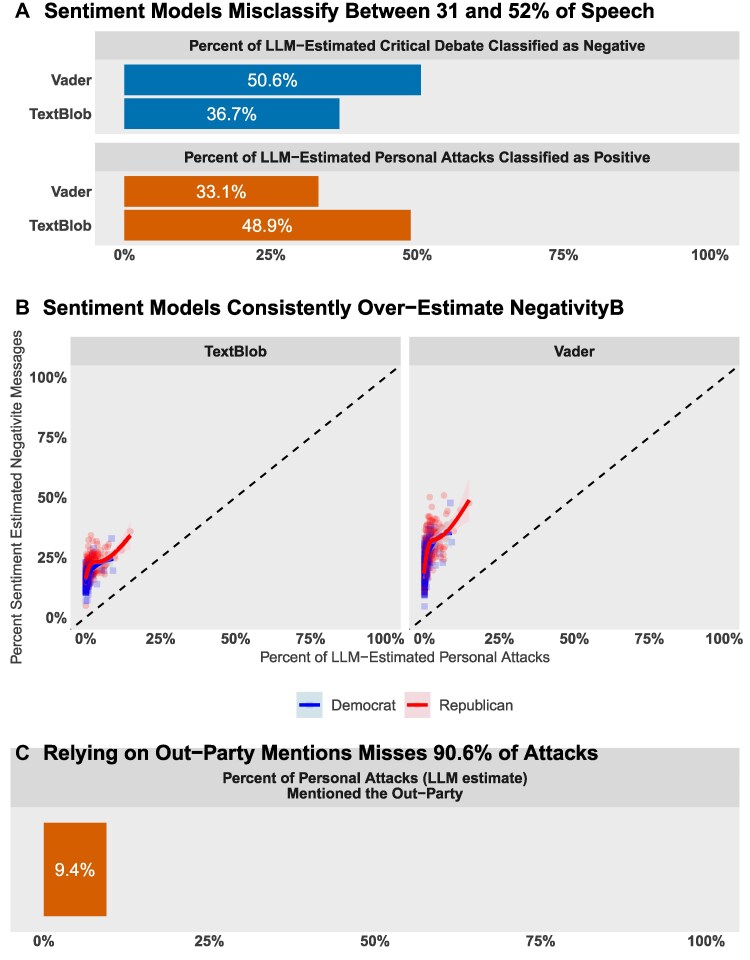
Our LLM-based measure outperforms standard methods for identifying personal attacks. Widely used sentiment classifiers (Vader, TextBlob) misclassify much critical policy debate as negative (A) while failing to detect the negativity of many personal attacks (B). An alternative approach, flagging mentions of the opposing party, misses the vast majority of personal insults (C). This demonstrates the need for a context-aware method to distinguish substantive critique from personal invective. Loess curves and 95% CI.

The LLM classified text across several dimensions (see Fig. 1 and SI Section 2), of which we analyze three: (i) personal attacks, (ii) critical debate, and (iii) policy discussion. We define personal attacks as statements targeting the character, integrity, intelligence, or morality of individuals or political groups, while specifically excluding critiques of policy. By contrast, negative discussion of policy without personal targeting was classified as critical debate.

## Conflict entrepreneurship in the 118th congress

### Who are conflict entrepreneurs?

**Fig. 2.**
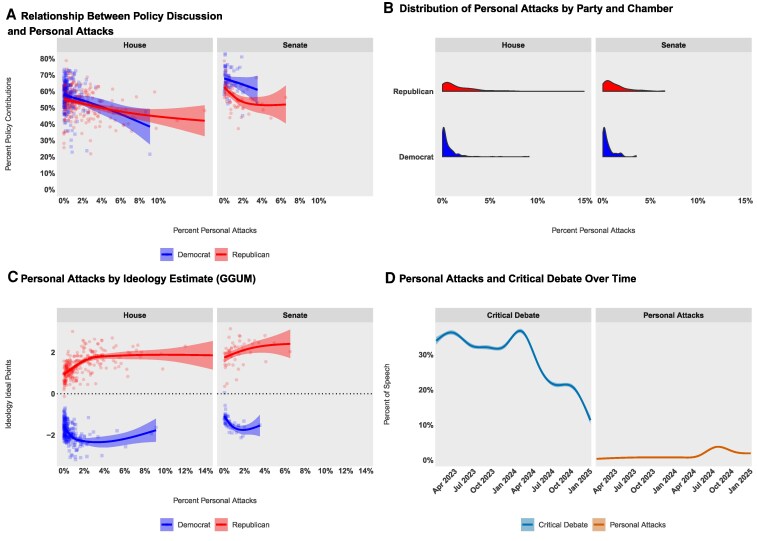
The percentage of personal attacks increases as policy discussion decreases (A). Most legislators minimally deploy personal attacks, though there are outliers in both parties and chambers (B). Personal attacks increase as legislators become more ideologically extreme, though this relationship is less pronounced as ideology increases to the extremes (C). Personal attacks increase in the period before an election growing from a weekly average of ∼0.2% to a high of ∼5.5%, while critical debate is considerably more common overall, it plummets as elections approach from a weekly high of ∼42.1% to a low of ∼7.2% (D). Loess curves and 95% CI are shown in (A), (C), and (D).

We theorize that legislators face a tradeoff between focusing on substantive policy and emphasizing partisan divisions. Our data support this: the more frequently legislators engage in personal attacks, the less likely they are to discuss policy. Figure 2A shows a clear negative relationship between the share of a member’s communications devoted to personal attacks and the share devoted to policy discussion. This pattern, consistent across both parties and chambers, provides evidence that personal attacks often come at the expense of substantive debate. This aligns with our theoretical framework, which suggests that because conflictual language draws disproportionate media attention, some legislators will choose to concentrate their rhetorical effort on personal attacks rather than on policy advocacy (23, 24, 26).

Although conflict entrepreneurship appears in both parties and chambers, it remains an infrequent strategy for most members of Congress. As shown in Fig. 2B, 35 legislators (6.4%) never employ personal attacks, and 352 legislators (65.0%) use them in <1% of their communications. A small minority, however, consistently allocates a substantial share of their rhetoric to personal insults.

Republican members make personal attacks at higher rates than their Democratic counterparts in both the House and the Senate. Nonetheless, the vast majority of Republicans continue to prioritize policy discussion over personal attacks. Conflict entrepreneurship is also more common in the House than in the Senate, reflecting the more adversarial norms and faster news cycles of the lower chamber.

Ideological extremism is associated with greater conflict entrepreneurship (see Fig. 2C). In both chambers, more conservative Republicans engage in conflict entrepreneurship at higher rates than their more moderate peers. The pattern among Democrats is more nuanced, as there is an initial increase in personal attacks with an increase in ideological extremity, but in both chambers the most extreme members are less likely to make personal attacks than the modal Democrat. These descriptive patterns align with research linking ideological extremity and affective hostility to the use of negative rhetoric (17). The asymmetry in this relationship—stronger among Republicans than Democrats—reflects broader patterns of asymmetric polarization in contemporary American politics, where both parties have shifted but with different magnitudes and at different rates.

Conflict entrepreneurship also varies over the electoral cycle. Figure 2D shows that personal-attack rates remain low and stable in nonelectoral periods but rise sharply in the months preceding general elections, mirroring established patterns of campaign negativity (1, 12). The weekly mean of personal attacks is 1.3% (SD = 1.1%; range = [0.2%, 5.5%]). Consistent with earlier findings, there are only 22 instances where a legislator made exclusively personal attacks during a week (17 unique legislators), and every legislator has gone at least 1 week without making a personal attack.

By contrast, the frequency of critical debate fluctuates more widely, peaking when Congress adjourns and as campaigns intensify. Critical debate also never recovers in the period following an election. A total of 530 legislators have gone at least 1 week without engaging in any critical debate (weekly mean = 28.9%; weekly SD = 8.3%; range = [7.2%, 42.1%]). The sharp decline in critical debate observed between early 2024 and 2025 (Fig. 2D) represents a genuine shift in congressional discourse rather than a methodological artifact. We verified that no changes in data collection or classification procedures occurred during this period. Two factors likely explain this pattern: First, the 2024 election cycle may have shifted rhetoric from policy criticism to electoral messaging and personal attacks. Second, the legislative calendar during this period featured fewer substantive bills under debate, reducing opportunities for policy-focused criticism. This pattern underscores how institutional context and electoral timing shape the balance between substantive and personal rhetoric in Congress.

### Personal attacks occur in both formal and informal settings

While some members consistently rely on conflict, they often tailor their level of negativity to the venue. Personal attacks are more than three times as common on X (1.8% of posts) than in the formal setting of the House or Senate floor (0.5% of speech segments). Press releases (1.1%) and newsletters (0.6%) fall in between. This suggests representatives are more aggressive when communicating directly with the public or media and more reserved in procedural forums or those with narrower audiences (30, 49).

**Fig. 3.**
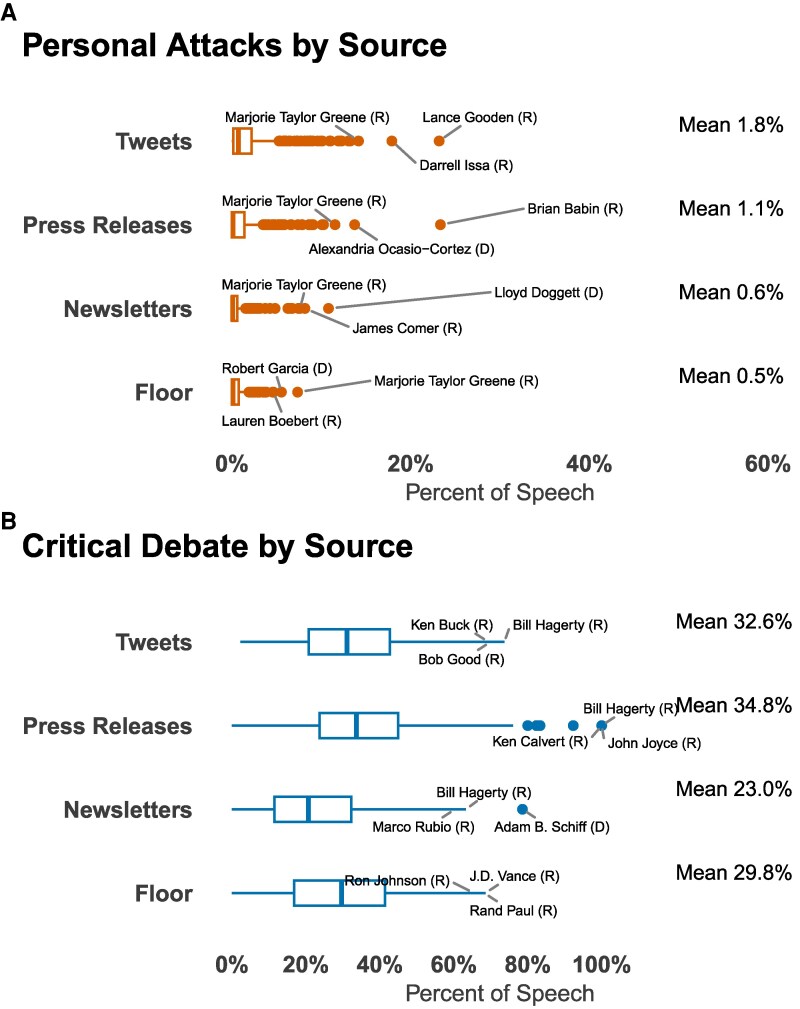
These are box plots that show the prevalence of rhetoric by source. Most legislators do not make many personal attacks, but there are extreme outliers, with some attacking at more than 10 times the overall mean. Attacks are also three times more common on social media than on other sources (A). In contrast, most legislators engage in ample critical policy debate (B). The box shows the IQR, the bold vertical line indicates the median and the horizontal lines represent the largest value observed before 1.5 times the IQR plus the value of the third quartile. The points are outliers.

As Fig. 3A shows, a small subset of legislators are outliers, dedicating over 20% of their social media posts or press releases to personal attacks. By contrast, most members allocate roughly one-third of their rhetoric to substantive, critical policy debate across all channels (Fig. 3B).

### Who is attacked?

Members of Congress consistently target the head, or perceived head, of the opposing party, as well as the parties themselves. Table 1 reports those targets with more than 150 occurrences; overall, we identify 43,560 instances in which a person or group was attacked. The three presidential candidates in the 2024 cycle account for 14,434 mentions (33.1% of total attacks), while the two major parties receive 1,393 mentions (3.2%). Remaining attacks focus on other high-profile actors, including individual legislators (e.g. Senator Ted Cruz; Representative Jim Jordan), judicial figures (e.g. Justice Thomas), and executive officials (e.g. Secretary Mayorkas). This concentration of rhetorical attacks on high-salience figures underscores the strategic nature of congressional discourse and its role in structuring public attention to partisan competition.

**Table 1.** The most attacked people and groups.

Target	*N*
Joe Biden	8,546
Kamala Harris	2,958
Donald Trump	2,930
Democrats	779
Republicans	614
The Judiciary	537
Mayorkas	411
Tim Walz	321
Elon Musk	154
Fauci	154
Total	17,553

Democratic presidential contenders received the lion’s share of rhetorical assaults—approximately four times the number leveled at President Donald Trump. In contrast, when we restrict our analysis to congressional targets, Republican members of both the House and the Senate are subjected to more frequent partisan invective than their Democratic colleagues. These differences are likely in part related to the constellation of majorities in the two chambers and the incumbent president. Our data span the 118th Congresses, during which House control shifted to Republicans while the Senate and presidency remained under Democratic majority control. Partisan attacks may thus also partially reflect institutional power dynamics, as opposition-party members have greater incentives to attack incumbent politicians or the majority party, while majority-party legislators have more incentives to justify and defend their decision-making or target members in the other chamber that is under control of the out-party.

## Relationships to outcomes

### Media attention

Conflict entrepreneurs receive disproportionate media attention. As predicted by the attention hypothesis, which posits that politicians use incivility to attract media coverage, we find a strong positive correlation between the use of personal attacks and media mentions.

**Fig. 4.**
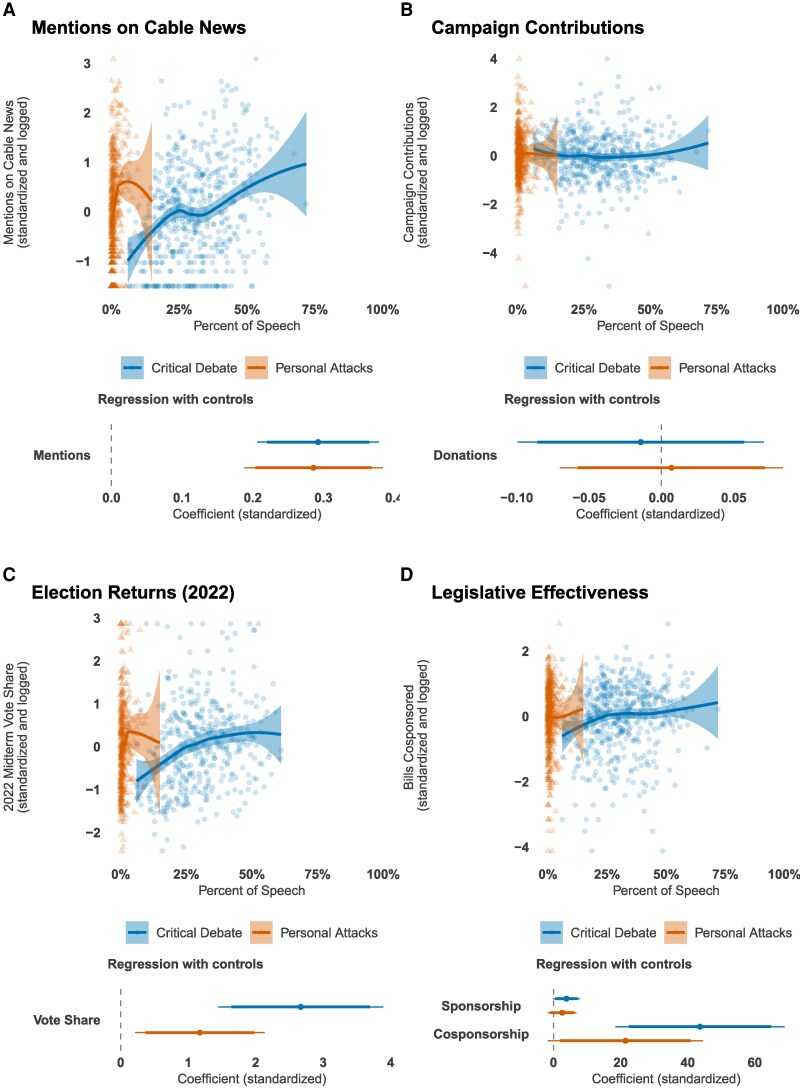
Personal attacks are related to more media attention (a legislator with a ∼5% share of personal attacks is associated with the level of media attention expected for a legislator with a ∼45% share of critical debate) (A), are not related to campaign contributions (B), and are only weakly related to electoral returns (C). Loess curves and 95% CI are shown in the top (A–D). In the bottom, we show standardized regression estimates with 90% and 95% CI. The models include time in office, party, chamber, gender, district competitiveness (PVI), and ideological extremity (absolute value of the GGUM score) as controls. We report ordinary least squares (OLS) regression models with region and state fixed effects in SI Section 4.

This relationship is particularly evident in cable news coverage. Legislators with a higher share of personal attacks in their communications receive levels of media attention comparable to those who dedicate a much larger share of their rhetoric to critical debate. For instance, a legislator who devotes 5% of their communication to personal attacks receives a similar amount of media attention as a legislator who dedicates 45% of their communication to critical policy debate (Figure 4A). For context, the 25 most conflictual members of Congress receive more cable news attention than the 75 least conflictual members combined.

Patterns on social media follow a similar trend. On X (formerly Twitter), posts that include personal attacks are, on average, shared far more often than posts featuring critical debate (mean of 606 vs. 244 retweets) and receive substantially more likes (mean of 2,369 vs. 760). These patterns indicate that greater personal attack intensity is associated with more immediate media visibility and broader audience reach across multiple channels.

### Fundraising

Research on negativity suggests that such language rallies a legislator’s base (12). However, evidence on its effect on donations, which primarily come from the base, is mixed (64, 65). We find no fundraising advantage associated with focusing on critical policy debate, nor is there a penalty for those who engage in personal attacks (Fig. 4B). Legislators who personally attack fellow politicians raise neither more nor less money than their more civil counterparts. This null relationship holds for both in-state and out-of-state donations.

### Electoral performance

Conflict entrepreneurship is correlated with electoral margins in the 2022 midterm elections (Figure 4C), with both more personal attacks and more critical debate leading to higher vote share. However, this bivariate relationship masks the underlying structural drivers of electoral success. Once we control for pre-election district competitiveness—operationalized using the Cook Partisan Voting Index (PVI) (66)—the coefficient on personal attacks becomes statistically indistinguishable from zero. In other words, conflict entrepreneurs are disproportionately located in less competitive districts and thus have higher margins of victory irrespective of their rhetorical style.^d^ Overall, our multivariate models provide no evidence that engaging in personal attacks yields a substantive electoral advantage.

Moreover, conflict entrepreneurship is neither necessary nor sufficient for electoral success: most candidates refrained from personal attacks, yet still secured between 40% and nearly 100% of the two-party vote share.

### Legislative efficacy

Conflict-driven legislators are less likely to serve on powerful committees, even after controlling for tenure in Congress. Although conflict entrepreneurs are no more or less likely to hold formal leadership positions (such as committee chairs or ranking memberships), they receive fewer assignments to prestigious standing committees, with the exception of senators, for whom no difference emerges (see SI Section 5 for full results). These patterns are consistent with theories of conditional party government, which emphasize centralized party control over committee assignments and the incentives for legislators to conform to party preferences (67).

Second, conflict entrepreneurship is not associated with either the introduction or cosponsorship of legislation. That is, personal attacks do not translate into greater substantive legislative activity or policy victories (22).^e^ By contrast, legislators who engage in critical yet policy-focused debate are significantly more likely to cosponsor bills, suggesting that civil policy engagement is a stronger indicator of formal legislative entrepreneurship.

### Changes in net worth

A long-standing public suspicion holds that legislators use their visibility to enrich themselves. If personal attacks function as a branding strategy, this might translate into lucrative opportunities. To test this, we examine the relationship between members’ rhetoric and year-over-year changes in their net worth, calculated from annual Ethics in Government Act financial disclosures for 2022–2023.

**Fig. 5.**
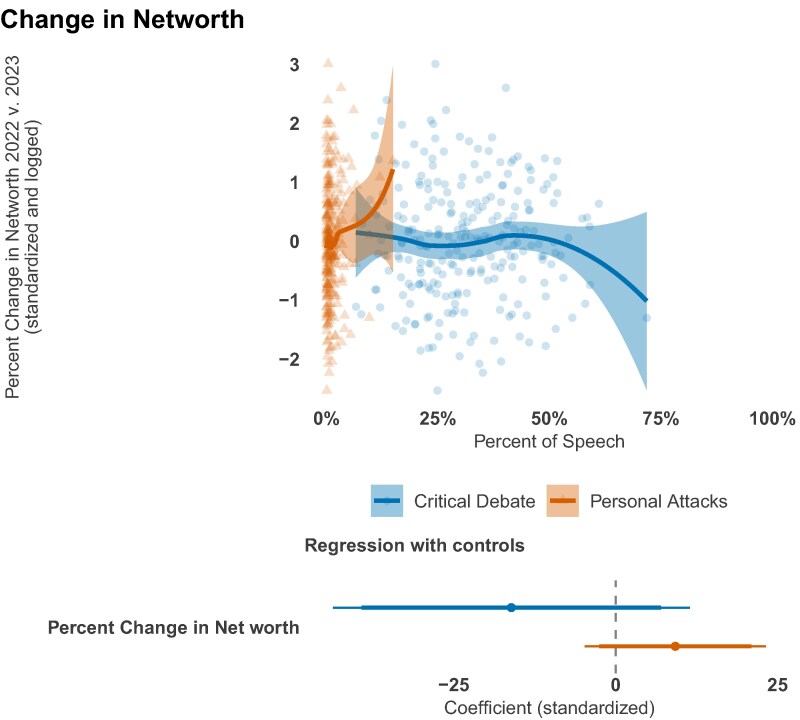
There is no relationship between either conflict entrepreneurship or critical debate and changes in net worth. Loess curves and 95% CI are shown in the top. In the bottom, we show standardized regression estimates with 90% and 95% CI and tenure, party, and chamber controls.

Following the methodology of Eggers and Hainmueller (68), we calculate each member’s net worth by taking the midpoint of reported asset and liability ranges. As Fig. 5 shows, the relationship between rhetorical style and percentage change in net worth is effectively nil for both personal attacks and critical debate. This null finding holds in multivariate models controlling for tenure, baseline wealth, party, and chamber (see lower panel of Fig. 5).

Our findings therefore suggest that during a legislative term, conflict entrepreneurship is a reputational rather than a pecuniary strategy. The visibility gained from personal attacks does not appear to translate into immediate, measurable wealth. This does not, however, preclude a longer-term financial motive. The brand recognition and media presence cultivated through a conflict-oriented style—“celebrity capital”—may be an investment in a postcongressional career, with potential payoffs from media contracts, lobbying positions, or consulting roles that materialize only after a member leaves office. Future research tracking legislators’ earnings over 5- to 10-year periods after leaving office would provide a more complete picture of the financial returns to vitriolic rhetoric. While testing this long-term hypothesis is beyond the scope of our current data, it suggests the financial incentives tied to political attention may operate on a timeline that extends well beyond a single term in Congress.

## Elite rhetoric vs. mass sentiment

**Fig. 6.**
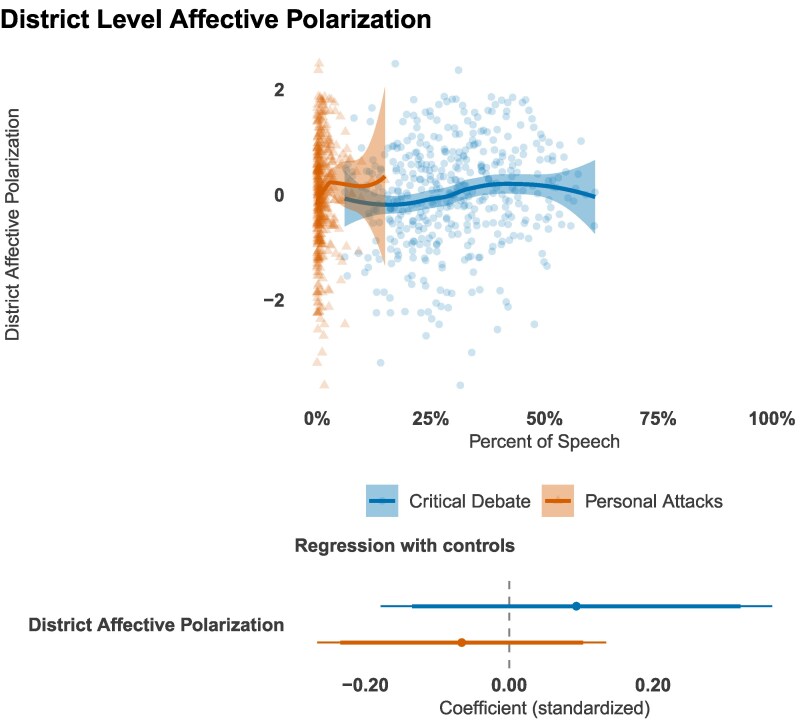
The most conflictual members are not from the districts most hostile to the other party. Out-group affect estimated with MRP. Weighted Loess curves and 95% CI are shown in the top. In the bottom, we show Deming regression estimates with 90% and 95% CI.

Do conflict entrepreneurs merely mirror the animus of the voters who elect them, or does their rhetoric exceed (and perhaps reshape) district-level sentiment? To adjudicate between these perspectives, we estimate *out-group affect* toward the opposing party for every congressional district using multilevel regression with post-stratification (MRP) applied to 140,000 interviews from the *America’s Political Pulse* survey.^f^ Figure 6 plots each legislator’s share of personal attacks and critical debate against the mean out-group feeling thermometer score in their district.

The results show a striking disconnect between elite rhetoric and mass sentiment. Two patterns are immediately apparent. First, the cross-sectional relationship between district hostility and a legislator’s use of personal attacks is effectively null. Many of the most abrasive legislators come from districts with comparatively moderate electorates, while many representatives from the most hostile districts use almost no personal insults. Second, the slope of the Deming-regression line, which accounts for measurement error in both variables, remains close to zero. This finding echoes a broader literature documenting misalignment between mass and elite opinion on ideology (69) and showing that politicians systematically misperceive their constituents’ views (70).

Why, then, do some members adopt a rhetorical style their districts neither demand nor reflect? One explanation is structural: as American politics has nationalized, legislators increasingly cultivate audiences beyond their home constituencies, including small-dollar donors, partisan media networks, and online activists who may favor more confrontational rhetoric (71, 72). A second possibility is perceptual: elite surveys reveal that officeholders routinely overestimate the extremism of their own electorates (70). A misperceived demand for incivility may therefore sustain its supply. We acknowledge, however, that our district-level estimates may blur the preferences of highly engaged subconstituencies—such as online activists or national small-dollar donors—who may be the true, nationalized audience for this rhetoric.’

Nevertheless, the patterns point to a troubling juxtaposition: personal attacks are frequently accompanied by elevated media attention even when the legislators employing them are out of step with their districts’ prevailing sentiments.

## Conclusion

Our analysis explores a central puzzle in contemporary American politics: the persistent supply of uncivil rhetoric despite citizen demand for substantive, respectful political discourse. The answer lies not in electoral incentives or financial rewards, but in the media attention economy that rewards conflict over substance. Normatively, legislators who advance substantive policy debates ought to be rewarded, whereas those who rely on personal attacks should incur electoral or institutional sanctions. Empirically, however, we observe a different pattern. Personal attacks correlate with elevated media visibility, yet neither attack-oriented speech nor critical policy debate exhibits a statistically meaningful association with campaign-finance receipts, and both display only a modest—and nonrobust—positive correlation with subsequent vote share. We likewise find no systematic link between conflict entrepreneurship and changes in net worth, suggesting that reputational gains from incivility do not translate into private financial advantage.^g^ The absence of clear electoral, financial, or institutional penalties for uncivil rhetoric raises concerns about the capacity of democratic institutions to discipline norm-violating behavior.

Media outlets tend to emphasize insults and conflict over substantive discussion, creating what Iyengar and Kinder (73) term a “spotlight” incentive structure that appears to encourage conflict entrepreneurship. Under this incentive structure, legislators can cultivate a national audience without investing in legislative entrepreneurship, committee work, or policy specialization—activities traditionally associated with advancement in Congress (74, 75). This pattern reveals a fundamental shift in the objective functions of some legislators. While Mayhew (75) famously argued that members of Congress are “single-minded seekers of reelection,” and Fenno (74) identified the trinity of goals as reelection, influence within the chamber, and good public policy, our findings suggest a fourth objective has emerged: media celebrity.

The heterogeneity in legislator objectives has important implications for democratic accountability: institutional mechanisms designed to constrain legislators seeking reelection or chamber influence may fail to discipline those pursuing media visibility. While Republican legislators are among those who engage in incivility most frequently and, on average, issue more attacks than Democrats, a non-negligible number of Democratic legislators also engage in ad hominem attacks. Overall, however, in both parties, the vast majority of legislators prioritize policy debate over personal attacks.

Our findings support the attention hypothesis proposed by Zeitzoff (12), yet they challenge several common assumptions about political negativity:

1. **Prevalence.** While *explicit* personal attacks captured by our text-based methodology are comparatively rare, they receive a disproportionate share of media coverage and social media engagement, distorting the public’s perception of congressional discourse. Our estimates likely represent a lower bound, as implicit attacks, visual content, and coded language are not captured by our approach.
2. **Timing.** This rhetoric is not confined to election season. While personal attacks intensify before an election, they remain a consistent feature of discourse even in nonelectoral periods, confirming the “permanent campaign” thesis that American politics has entered a state where governing and campaigning are no longer distinct phases (50). Notably, we observe a sharp decline in substantive policy debate during the 2024–2025 transition period, suggesting that electoral cycles and legislative calendars jointly shape the balance between critical policy discussion and personal attacks.
3. **Electoral payoffs.** Contrary to the beliefs of many practitioners (18), conflict entrepreneurs do not enjoy systematically higher electoral returns than their policy-minded colleagues once partisanship is controlled for.
4. **Opportunity costs.** Engaging in personal attacks comes at the expense of policy discussion, indicating that uncivil rhetoric substitutes for, rather than complements, substantive debate.
5. **Private gain.** We find no relationship between personal attacks and *short-term* changes in members’ net worth while in office. However, our 1-year wealth measure cannot capture the potentially substantial financial returns from brand-building that may materialize as lucrative media careers, speaking engagements, or book deals after leaving Congress. Conflict entrepreneurs may be rationally investing in controversial personas that yield deferred compensation exceeding what policy-focused legislators can command in postcongressional careers.
6. **Constituent alignment.** The use of personal attacks shows little correspondence with constituent animosity, underscoring a mismatch between elite rhetoric and mass sentiment.

While these findings overall indicate that conflict entrepreneurs do not derive tangible returns from their incivility, there may be less visible benefits to engaging in such behavior. From a psychological perspective, conflict entrepreneurs may generate satisfaction from the mere attention their attacks produce. Moreover, attacks may be a resource in party organization in which such behavior may be an expression of loyalty to the leadership; in the case of the current Republican Party, in this logic, attacking others personally may be perceived as a loyalty signal to Republican President Trump, who regularly issues such attacks on opponents as well.

A key caveat of our analysis is that the evidence presented here is descriptive. We do not know whether the same legislators would have fared differently absent their conflict-oriented communication strategies. Nevertheless, by documenting the prevalence, correlates, and institutional positioning of conflict entrepreneurs, we furnish a foundation for future causal work—whether through field experiments, panel methods, or quasiexperimental shocks—that can more precisely adjudicate the strategic calculations animating elite rhetoric.

If left unchecked, conflict entrepreneurship may erode democratic accountability. While the mass public expresses distaste for personal attacks, they are often unaware of the specific legislators who deploy them. Strong, programmatic parties could mitigate this problem by withholding leadership positions, committee assignments, or campaign resources from frequent violators (76). Likewise, journalistic gatekeepers can improve democratic discourse by contextualizing incendiary remarks rather than amplifying them as entertainment, much as sports broadcasters frame fights in the National Hockey League.

## Methods and data

### Policy contributions

In addition to constructing a personal attack measure for members of Congress, we classify references to policy in their political statements. Specifically, we present the AI model with a prompt that requests the classification of all statements as policy contributions if the text includes discussions on policy, encompassing specific legislation or general discussions on topics such as healthcare, education, environment, foreign policy, the economy, defense spending, national security, etc. The prompt further provides an extensive list of policy areas, each with definitions, covering a wide range of subjects from agriculture and food to water resources development.

### Media appearances

We use the written transcripts of major US cable TV channels (CNN, MSNBC, and FoxNews) and count how often each legislator is mentioned on air. The number of mentions provides us with a measure of media visibility, that is, the degree to which media pays attention to legislators and, in particular, to conflict entrepreneurs.

### Campaign contributions

We use data from the Federal Election Commission to record the sum of donations and the number of donors for each member of Congress. We examine the relationship between raising donations and members of Congress’ rhetoric, separately for in-state and out-of-state donations and donors. Lastly, we calculate the ratio of in-state to out-of-state donations and donors, which allows us to discern what types of legislators are more likely to have a national rather than a regional donor support base.

### Election returns

We use data from the 2022 midterm elections to measure the most recent electoral performance for each member of Congress. We restrict this analysis to members of the House of Representatives only because all members of the House are on the same election cycle.

### Ideology

We estimate members of Congress’ ideological orientation using generalized graded unfolding model (GGUM) ideal point estimation (78). Based on each legislator’s voting record in the 118th Congress, we implement an ideal point estimation approach that takes into account that more extreme legislators tend to reject moderate bills.

### Committee rank and assignment

We collect data on members of Congress’ committee ranks and assignments. These data allow us to evaluate whether conflict entrepreneurs are more or less likely to be assigned to serve as committee chairs or ranking members and whether they are assigned to committees with more policy impact. To account for the potential role of seniority in committee ranks and assignments, we implement linear probability models controlling for years served in Congress.

### Changes in net worth

Annual personal–financial disclosures filed under the *Ethics in Government Act* serve as the basis for our net-worth data. We scraped the electronically filed reports for calendar years 2022 and 2023 from the Clerk of the House and the Secretary of the Senate websites, then matched them to member identifiers in our speech corpus.^h^ Following Eggers and Hainmueller (68), we convert the reported asset and liability ranges to dollar values by taking the midpoint of each interval and summing across all entries. Spousal holdings are included when listed; qualified retirement accounts and defined-benefit pensions, which are reported without value ranges, are omitted.

### District out-group affect

We estimate district-level hostility toward the opposing party with MRP. The survey input is the *America’s Political Pulse* study, fielded for 138 weeks starting in 2022. Data include US adults drawn from the YouGov panel. Respondents rated Democrats and Republicans on separate 0–100 feeling thermometers; we define *out-group affect* as the thermometer rating of the respondent’s nonpreferred party, rescaled so higher values indicate greater dislike.

## Supplementary Material

pgag038_Supplementary_Data

## Data Availability

All data and scripts necessary to replicate the analysis in this article are currently on OSF: https://osf.io/v5nr8/?view_only=82213dd48220449eaceba9b41649c4d2

## Notes

a Marjorie Taylor Greene, R-GA, Tweet, https://twitter.com/RepMTG/status/1565515860466343938?lang=en.

b Jared Moskowitz, D-FL, https://twitter.com/JaredEMoskowitz/status/1755765945123881365.

c X posts were analyzed in their entirety due to their character limits.

d We further subdivided districts into competitive (PVI within ±5 points), semicompetitive (PVI between ±5 and ±15 points), and safe (PVI beyond ±15 points) categories and found no systematic relationship between conflict intensity and electoral performance at any competitiveness level (see Fig. S5).

e These results also hold for bill passage, although the low number of enacted measures in the 118th Congress limits the statistical power of this test.

f The MRP strategy follows the procedure in Lax and Phillips (77), with post-stratification to the Census 2020 5-year American Community Survey.

g Our analysis is limited to financial changes during congressional service. We cannot observe whether conflict entrepreneurs command higher speaking fees, book advances, or media contracts after leaving office—potentially substantial financial incentives that would not appear in our data.

h Filers who entered or exited office midcycle are excluded because the reports do not cover a full year.
