# Supplementary material for: Entrepreneurs of conflict: A descriptive analysis of when and how political elites use divisive rhetoric
Source: PNAS Nexus. 2026 Mar 17;5(3):pgag038. doi: 10.1093/pnasnexus/pgag038 (PMC12993816; doi:10.1093/pnasnexus/pgag038)
Supplement: pgag038_Supplementary_Data [file pgag038_supplementary_data.pdf]

Supporting Information

Entrepreneurs of Conflict: A Descriptive Analysis of When and How Political Elites Use Divisive Rhetoric

Marc S. Jacob* Yphtach Lelkes† Sean J. Westwood‡

February 17, 2026

Contents

1 Model Performance **2**

2 Classification scheme **2**
2.1 Prompts 2
2.1.1 Prompt text 2
2.2 Example classifications 4
2.2.1 Examples of Personal Attacks 4
2.2.2 Examples of Constructive Debate 5

3 Additional descriptive statistics **8**

4 Covariate analysis **13**
4.1 Policy and ideology 13
4.2 Election and fundraising performance 13
4.3 Media mentions 31
4.4 Legislative effectiveness 33
4.5 Net Worth 35
4.6 Constituent Preference 37

5 Committee assignment **39**

6 Affect (MRP) **45**
6.1 Multilevel Regression Model 45
6.2 Synthetic Poststratification Frame 45
6.3 Poststratification 45

*Stanford University.
†UPenn.
‡Dartmouth College.

Contents

1 Model Performance

	Accuracy	Precision	Recall
Trained annotators	92%	56%	82%
GPT 4o	97%	98%	92%

Table S1: Classification performance for personal attacks.

	Accuracy	Precision	Recall
Trained annotators	80%	83%	91%
GPT 4o	81%	84%	92%

Table S2: Classification performance for critical debate.

2 Classification scheme

2.1 Prompts

We developed a prompt to classify texts from elected officials. Prior drafts of this paper used an older prompt. We altered the prompt to its present form and reprocessed all data.

There are several things to note:

- First, prompts can be used by humans, but should not be seen as the equivalent of instructions provided to a human. We started with prompts we provided to humans in pre-testing and then added and removed components until we archived consistent output from the model.
- This means that some concepts are mentioned without explanation. Including definitions did not increase accuracy, so the definitions were removed to reduce the number of prompt tokens.
- We must also include instructions to guide the model that a human might not need. For example, we must tell the model to "not count attacks on companies or groups, foreigners, or terrorist groups" when this would likely be implicit to a human.

2.1.1 Prompt text

Evaluate the provided text, with JSON results. Evaluate each of the following tasks sequentially.

1. **Attacks**

- **Task:** Assess the text for `personal_attack` (output either "yes" or "no"). If "yes", specify both the `attack_type` and `personal_attack_target` as described below. If "no", set `attack_type` and `personal_attack_target` to `null`.
- **Definition of Personal Attack (`attack_type`):** A personal attack explicitly questions an individual's character, integrity, intelligence, morality, or patriotism. Opposition to an individual or their policy choices does not count. Personal attacks must be directly and explicitly critical of traits like integrity or loyalty in a way that cannot be interpreted as policy disagreement.
  - *character*: Calling someone lazy, unreliable, stupid, etc.
  - *integrity*: Accusing someone of dishonesty, lying, embellishing the truth.
  - *intelligence*: Claiming someone is mentally unfit or incompetent.
  - *morality*: Accusations of corruption, unethical behavior, or criminality.
  - *patriotism*: Accusing someone of being un-American or anti-democratic.
- **Target Identification (`personal_attack_target`):** Record the name of the person attacked (no titles, up to two words, e.g., "Joe Biden").
- **Exclusion criteria:** Do not consider statements as personal attacks if they:
  * Critique job performance, policies, legislation, or appointments without explicit character attacks.
  * Require assumptions or interpretations.
  * Question leadership decisions or support for U.S. policy.
  * Are quoted or general references (e.g., "attackers").

2. **Policy Criticism**

- **Task:** Assess the text for `policy_attack` (output either "yes" or "no"). If "yes", the text must object to a specific policy, law, or court ruling with fact-based critique, and must not overlap with personal attacks.
- **Definition:** A constructive critique of policy, avoiding emotional or extremist language and excluding cases where `personal_attack` is "yes".

JSON Output Format

```
{
  "attacks": {
    "personal_attack": ,
    "attack_type": [],
    "personal_attack_target":
  },
  "policy_criticism": {
    "policy_attack":
  }
}
```

```
Text: "{target}"
```

2.2 Example classifications

In the tables below we provide 10 examples and model explanations. These are a random sample of items.

2.2.1 Examples of Personal Attacks

Table S3: Examples of Personal Attacks

bioguide_id	Text	Model Explanation
B001243	Senate Democrats BLOCKED my American Girls in Sports Day resolution to celebrate the accomplishments of real women in the athletic space. Democrats pretend to champion women until they're in a man's way.	The text criticizes Democrats by questioning their integrity and claims they only support women selectively, which constitutes a personal attack.
C001080	FAILED: Speaker Johnson's concept of a plan to fund the government just went down in flames. He should stop taking his Project 2025 marching orders from Trump and start working on a bipartisan basis to deliver for Americans.	The text explicitly criticizes Speaker Johnson's character by implying he follows Trump blindly instead of working constructively.
M001137	Republicans are playing political games with our nation's economy. We must pass a clean CR that does not tack on frivolous measures that cheat veterans and increase Social Security Administration wait times. Democrats are ready to work across the aisle	The text accuses Republicans of playing political games with the nation's economy, questioning their integrity.
S001176	BREAKING: 158 House Democrats just voted AGAINST requiring the deportation of illegal immigrants who assault women in the United States. This is who they are.	The text explicitly attacks the Democrats by questioning their morality, suggesting they support illegal immigrants who assault women.
S000148	For Donald Trump to suddenly pretend while he's speaking on Long Island that he's found religion on eliminating the SALT caps is comical and unserious. His promises carry as much weight as monopoly money.	The text attacks Donald Trump's sincerity and credibility, questioning his character by suggesting his promises are worthless.

Continued on next page

Table S3 – continued from previous page

bioguide_id	Text	Model Explanation
C001095	Our allies know that Joe Biden and Kamala Harris can't be trusted with vital national security information. Rather than support Israel, Biden-Harris officials are throwing a hissy fit and leaking supposed details to the media.	The text attacks Joe Biden and Kamala Harris's integrity and reliability regarding handling national security information, suggesting they can't be trusted.
S001193	Forgive Trump. He's almost 80. He's going to keep having slips. If president, for example, he will likely say "bomb Toronto" when he really wanted to say "bomb Tehran." These are just mistakes we have to accept from a man in a declining mental state.	The text explicitly questions Trump's mental state, implying a decline that affects his decision-making ability, which constitutes a personal attack.
S001195	The left wants you to believe that President Trump is to blame for the bomb hoaxes in Springfield, Ohio. Yet they insist their rhetoric calling President Trump the greatest threat to democracy for 4 years had nothing to do with two assassination attempts.	The text accuses Democrats of deflecting blame and insists their rhetoric may have incited assassination attempts, questioning their integrity.
A000371	House Republicans are once again leading us towards a government shutdown that will hurt children, veterans, and our military. We need to put an end to the chaos and dysfunction.	The text accuses House Republicans of causing chaos and dysfunction, questioning their integrity and competence. This is an explicit and overt attack on their character.

2.2.2 Examples of Constructive Debate

Table S4: Examples of Constructive Debate

bioguide_id	Text	Model Explanation
W000802	Backstage maneuver by the Chief Justice has been a telltale behind the worst Supreme Court political decisions. This article shows his maneuvering to steer the three insurrection-related decisions in Trump's favor.	The text critically discusses Supreme Court decisions related to insurrections but does not use personal attacks or emotional appeals.

Continued on next page

Table S4 – continued from previous page

bioguide_id	Text	Model Explanation
C001056	The Biden-Harris admin is playing a shell game w/stats on migrant encounters. Last night, the Senate UNANIMOUSLY passed my bill to hold them accountable. Dems knew they couldn't block legislation showing Americans how many migrants are being released on Kamala Harris' watch.	The text critiques the Biden-Harris administration's handling of migrant encounters, indicating they manipulate statistics, without making personal attacks.
C001056	Even with $1 Trillion a Year the US Military Is Falling Behind	The text constructively criticizes military spending policy, suggesting inefficiency despite large investment, without personal attacks.
D000563	Today, I sent letters to @uspto & 3 drug companies highlighting errors in patent term adjustment. Big Pharma benefits from these errors by extending their patent monopolies and delaying competition from lower-cost generics.	The text criticizes errors in patent term adjustments and their impact on competition without attacking individuals or political parties.
W000805	I'm still deeply disappointed that Republicans blocked a vote that would have codified access to IVF yesterday. Failing to protect IVF is a failure to protect our most private and fundamental liberties. I'm staying in this fight.	The text criticizes Republicans blocking legislation on IVF, focusing on policy failure not personal attacks.
B001267	Forty years of trickle-down economics has failed our middle class. I joined @RepLloydDoggett & @RepGwenMoore today to turn the page in 2025 to expand tax credits for working families, and put money back in the pockets of the people who need it most.	The text criticizes 'trickle-down economics' as having failed the middle class, which is a critique of economic policy rather than a personal attack.
B001236	Auto dealers in Arkansas and I agree the Biden administration's onerous EV mandate will harm consumers. I appreciated hearing their concerns with it and other regulations. We appreciate their advocacy and dedication to offering the cars and trucks customers desire. #ARinDC	The text constructively criticizes the Biden administration's EV mandate and regulations as harmful to consumers without using emotional appeals or personal attacks.

Continued on next page

Table S4 – continued from previous page

bioguide_id	Text	Model Explanation
C001047	Even with the cut, interest rates are still higher than any time since @realDonaldTrump was in the White House. Bottom line: The actions of Congressional Democrats and the Biden-Harris administration have long-term consequences and the American people are bearing their brunt.	The text criticizes the actions of Congressional Democrats and the Biden-Harris administration, attributing long-term negative consequences to their policies.
C001069	The short-term spending bill that the House voted down tonight would have harmed submarine construction, military families, and veterans. Now that it has been rejected by both sides of the aisle, Speaker Johnson must move forward with a bipartisan package to fund the government.	The text criticizes the short-term spending bill for negatively impacting submarine construction, military families, and veterans, focusing on policy outcomes.
D000197	They need to get serious right now about funding the government, which is Congress' most basic responsibility.	

3 Additional descriptive statistics

Most extreme conflict entrepreneurs. Who are the most extreme conflict entrepreneurs in Congress? Figure S1 lists the top 20 conflict entrepreneurs for each party. Among Democrats, Representatives Eric Swalwell, Robert Garcia, and Bill Pascrell Jr. attack most often relative to making substantive policy contributions. Among Republicans, Representatives Marjorie Taylor Greene, Clay Higgins, and Darrell Issa are the most extreme conflict entrepreneurs.

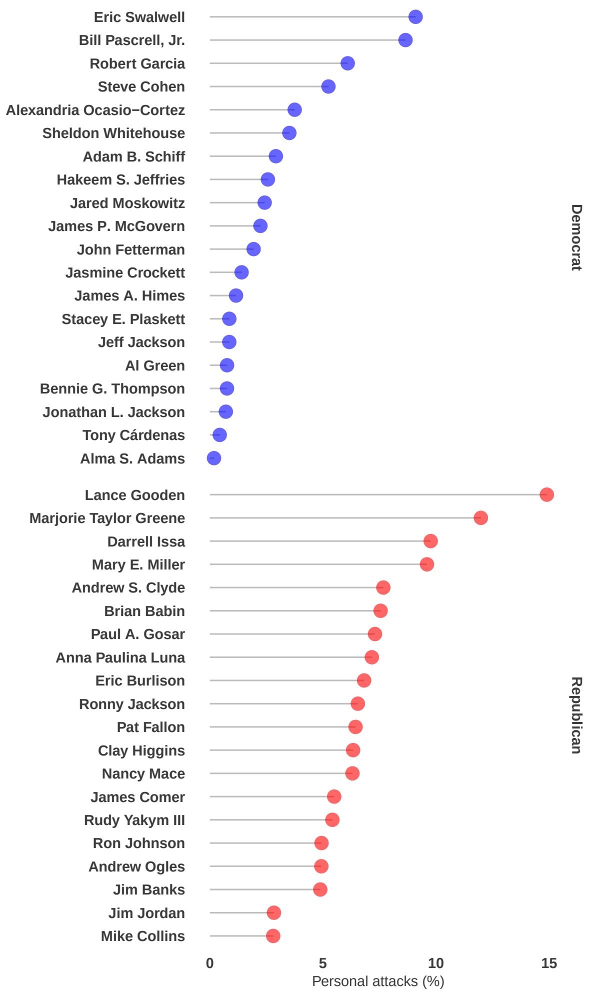

Figure S1: Top 20 personal attack scores for each party.

Geographic distribution. Figure S2 displays the average personal attack (Panel A) and critical debate (Panel B) scores by state. While there is no apparent pattern between states for critical debate, personal attacks are most common among legislators from three states: Georgia, Arizona, and Tennessee.

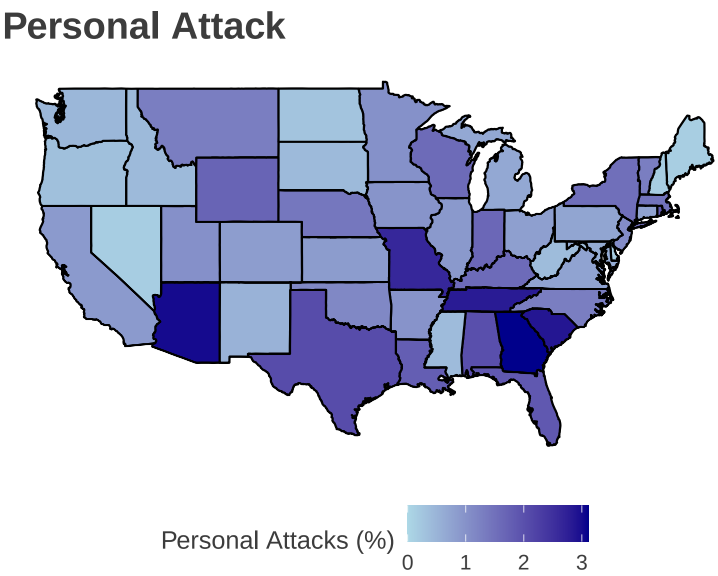

[Personal attacks]

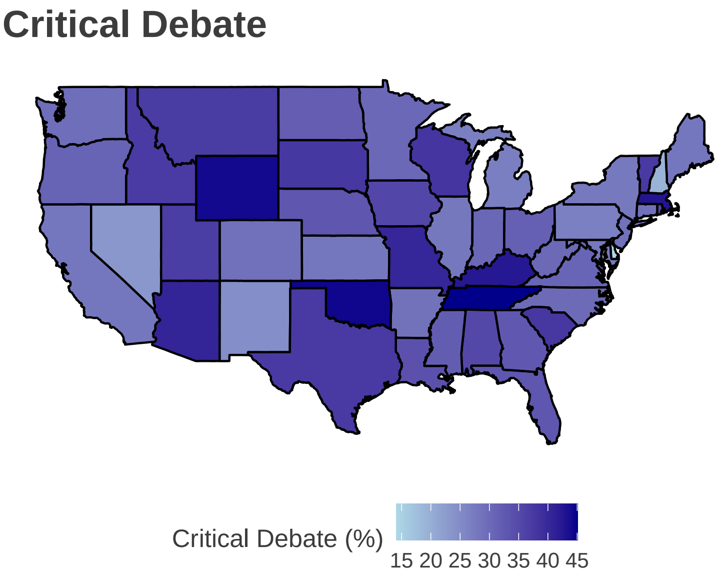

[Critical debate]

Figure S2: Average attack and critical debate scores by state.

Covariate relationships by party. Figures S3 and S4 show the relationship between personal attacks and critical debate, respectively, and media mentions, donations, and election returns.

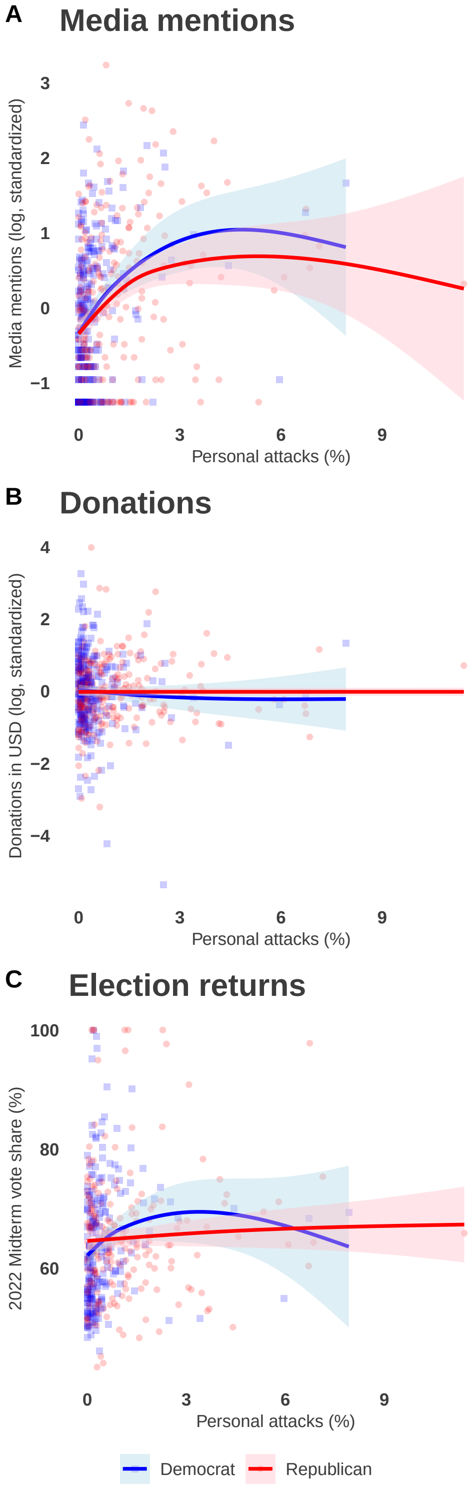

Figure S3: Relationship between personal attacks and covariates by party.

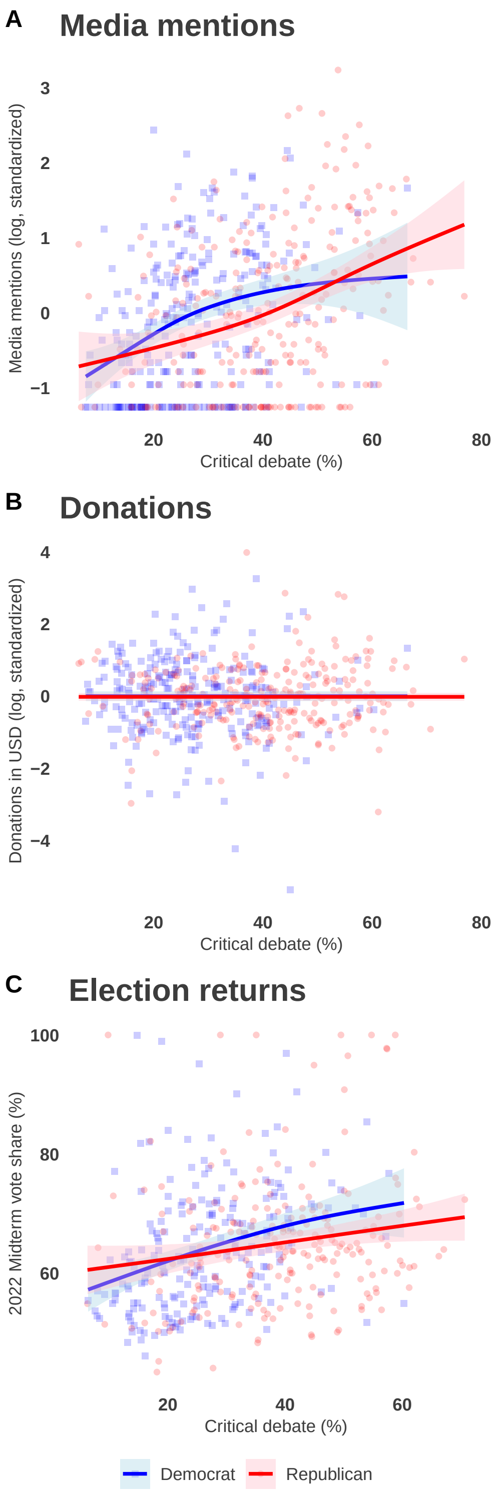

Figure S4: Relationship between critical debate and covariates by party.

4 Covariate analysis

This Appendix presents the results of regression models testing the relationship between personal attacks and the range of outcome variables outlined in the main text. Across all models, we implement the following regression models:

$$y_i = \beta_0 + \beta_1 x_i + \mathbf{X}\boldsymbol{\beta} + \delta x_i + \epsilon_i, \quad (1)$$

where y_i is the outcome variable of interest, $\beta_1 x_i$ a parameter for legislator i's personal attack score, $\mathbf{X}\boldsymbol{\beta}$ a vector of parameters of control variables. δx_i are fixed effects for either regions or states.

The models include time in office, party, chamber, gender, district competitiveness (PVI), and ideological extremity (absolute value of the GGUM score) as controls.

4.1 Policy and ideology

Table S5 shows the relationship between personal attacks and frequency of engagement in policy. Table S6 displays linear models regression legislators' ideal points on personal attacks and other control variables.

4.2 Election and fundraising performance

Table S7 shows the result of a linear model regressing election outcomes in the 2022 midterm elections on the personal attack scale. Table S8 reports a similar regression with critical debate as a predictor.

Figure S5 furthermore shows the relationship between personal attacks and electoral performance by district competitiveness. We calculated district competitiveness using the 2023 Cook PVI dataset (`https://www.cookpolitical.com/cook-pvi/2023-partisan-voting-index/118-district-map-and-list`). We do not find that legislators with a higher personal attack score perform better in competitive, semi-competitive, or safe districts.

Table S9 reports the result of a linear model regressing the amount of in-state donations on the personal attack scale. Table S10 shows a similar regression with critical debate as a predictor.

Table S11 displays the regression coefficients for similar models with the amount of out-of-state donations as the outcome variable. Table S12 shows a similar regression with critical debate as a predictor.

Tables S13 and S15 report the results with personal attacks as a predictor for the number of in-state and out-of-state donors, respectively. Tables S14 and S16 report the results with critical debate as a predictor for the number of in-state and out-of-state donors, respectively.

Lastly, Tables S17 and S19 show the results for personal attacks of linear models for the ratio of in-state donations and donors to out-of-state donations and donors, respectively. Tables S18 and S20 show the results of linear models for critical debate for the ratio of in-state donations and donors to out-of-state donations and donors, respectively.

	Model 1	Model 2	Model 3
Personal attacks (%)	-0.015^{***}	-0.014^{***}	-0.014^{***}
	(0.003)	(0.003)	(0.004)
Years in Congress	0.000	0.000	0.000
	(0.001)	(0.001)	(0.001)
Republican (vs. Democrat)	-0.010	0.002	0.006
	(0.012)	(0.013)	(0.014)
Senate (vs. House)	0.046	0.030	
	(0.028)	(0.030)	
Gender (Male)	-0.031^{**}	-0.030^{**}	-0.027^{**}
	(0.010)	(0.009)	(0.010)
Competitiveness	0.000	0.001	0.001
	(0.001)	(0.001)	(0.001)
Ideological Extremity	0.006	0.007	0.007
	(0.009)	(0.010)	(0.010)
Region Fixed Effects		✓	
State Fixed Effects			✓
R^2	0.146	0.181	0.292
Adj. R^2	0.132	0.162	0.191
Num. obs.	440	440	440

$^{***}p < 0.001$; $^{**}p < 0.01$; $^{*}p < 0.05$

Table S5: OLS: Regressing constructive debate/policy debate on personal attacks. Robust standard errors in parenthesis.

	Pooled			Democrat	Republican
	Model 1	Model 2	Model 3	Model 4	Model 5
Personal attacks (%)	0.054***	0.051***	0.057***	−0.000	−0.000
	(0.014)	(0.014)	(0.015)	(0.000)	(0.000)
Years in Congress	−0.004	−0.004	−0.004	−0.000	0.000
	(0.003)	(0.003)	(0.003)	(0.000)	(0.000)
Republican (vs. Democrat)	3.223***	3.147***	3.107***		
	(0.067)	(0.072)	(0.073)		
Senate (vs. House)	0.394**	0.486***			
	(0.139)	(0.143)			
Gender (Male)	0.195***	0.207***	0.215***	−0.000	−0.002
	(0.057)	(0.057)	(0.056)	(0.000)	(0.002)
Competitiveness	−0.020***	−0.021***	−0.019***	−0.000***	0.000
	(0.003)	(0.003)	(0.003)	(0.000)	(0.000)
Ideological Extremity	0.292***	0.279***	0.234**	−1.000***	1.005***
	(0.086)	(0.084)	(0.085)	(0.000)	(0.005)
Region Fixed Effects		✓			
State Fixed Effects			✓	✓	✓
R^2	0.924	0.928	0.937	1.000	0.999
Adj. R^2	0.923	0.926	0.927	1.000	0.999
Num. obs.	440	440	440	213	227

$^{***}p < 0.001$; $^{**}p < 0.01$; $^{*}p < 0.05$

Table S6: OLS: Regressing ideal points on personal attacks. Robust standard errors in parenthesis.

	Pooled			Competitive	Semi	Safe
	Model 1	Model 2	Model 3	Model 4	Model 5	Model 6
Personal attacks (%)	-0.239	-0.291	-0.371	-0.419	-0.593	-0.708^{*}
	(0.250)	(0.262)	(0.277)	(0.264)	(0.403)	(0.356)
Years in Congress	0.249^{***}	0.248^{***}	0.228^{***}	0.130^{**}	-0.079	0.121
	(0.057)	(0.058)	(0.062)	(0.048)	(0.117)	(0.102)
Republican (vs. Democrat)	5.048^{***}	3.917^{**}	3.357^{*}	2.416^{**}	1.865	2.552
	(1.229)	(1.319)	(1.360)	(0.754)	(1.736)	(2.884)
Gender (Male)	2.829^{**}	2.771^{**}	2.122^{*}	-0.054	0.993	1.208
	(0.993)	(0.967)	(1.037)	(0.786)	(1.865)	(1.885)
Ideological Extremity	7.507^{***}	7.270^{***}	7.054^{***}	1.585^{*}	-3.971	1.127
	(0.968)	(0.966)	(0.978)	(0.635)	(2.031)	(1.893)
Region Fixed Effects		✓				
State Fixed Effects			✓	✓	✓	✓
R^2	0.178	0.197	0.292	0.527	0.346	0.320
Adj. R^2	0.168	0.182	0.192	0.355	0.070	0.072
Num. obs.	429	429	429	140	139	143

$^{***}p < 0.001$; $^{**}p < 0.01$; $^{*}p < 0.05$

Table S7: OLS: Regressing 2022 midterm election results on personal attacks. Robust standard errors in parenthesis.

	Pooled			Competitive	Semi	Safe
	Model 1	Model 2	Model 3	Model 4	Model 5	Model 6
Critical debate (%)	0.023	0.048	0.063	0.022	−0.050	−0.005
	(0.060)	(0.060)	(0.062)	(0.040)	(0.111)	(0.088)
Years in Congress	0.248^{***}	0.243^{***}	0.222^{***}	0.122^{*}	−0.049	0.135
	(0.060)	(0.061)	(0.065)	(0.049)	(0.119)	(0.108)
Republican (vs. Democrat)	4.229^{**}	2.554	1.538	1.602	1.811	0.449
	(1.468)	(1.562)	(1.624)	(0.938)	(3.198)	(2.956)
Gender (Male)	2.800^{**}	2.732^{**}	2.077^{*}	0.040	0.616	1.232
	(0.996)	(0.971)	(1.041)	(0.786)	(1.897)	(1.968)
Ideological Extremity	6.888^{***}	6.274^{***}	5.749^{***}	0.865	−3.924	0.023
	(1.084)	(1.118)	(1.164)	(0.858)	(2.415)	(1.982)
Region Fixed Effects		✓				
State Fixed Effects			✓	✓	✓	✓
R^2	0.177	0.197	0.291	0.518	0.336	0.303
Adj. R^2	0.167	0.182	0.191	0.343	0.055	0.048
Num. obs.	429	429	429	140	139	143

$^{***}p < 0.001$; $^{**}p < 0.01$; $^{*}p < 0.05$

Table S8: OLS: Regressing 2022 midterm election results on critical debate. Robust standard errors in parenthesis.

	Pooled			Democrat	Republican
	Model 1	Model 2	Model 3	Model 4	Model 5
Personal attacks (%)	0.015	0.019	0.011	0.065	0.018
	(0.023)	(0.023)	(0.024)	(0.063)	(0.027)
Years in Congress	-0.019^{***}	-0.018^{***}	-0.019^{***}	-0.028^{***}	0.002
	(0.005)	(0.005)	(0.005)	(0.008)	(0.008)
Republican (vs. Democrat)	-0.281^{*}	-0.260^{*}	-0.190		
	(0.113)	(0.118)	(0.118)		
Senate (vs. House)	-0.851^{**}	-0.907^{**}			
	(0.283)	(0.277)			
Gender (Male)	0.018	0.029	0.051	-0.086	0.133
	(0.094)	(0.093)	(0.093)	(0.129)	(0.127)
Competitiveness	-0.011	-0.010	-0.013^{*}	-0.005	-0.029^{**}
	(0.006)	(0.006)	(0.006)	(0.008)	(0.010)
Ideological Extremity	-0.304^{**}	-0.304^{**}	-0.293^{**}	-0.521^{**}	-0.196
	(0.094)	(0.095)	(0.094)	(0.167)	(0.123)
Region Fixed Effects		✓			
State Fixed Effects			✓	✓	✓
R^2	0.129	0.136	0.302	0.349	0.458
Adj. R^2	0.115	0.115	0.202	0.187	0.322
Num. obs.	431	431	431	207	224

$^{***}p < 0.001$; $^{**}p < 0.01$; $^{*}p < 0.05$

Table S9: OLS: Regressing in-state donations (log) on personal attacks. Robust standard errors in parenthesis.

	Pooled			Democrat	Republican
	Model 1	Model 2	Model 3	Model 4	Model 5
Critical debate (%)	0.011^{**}	0.011^{*}	0.012^{**}	0.026^{**}	0.006
	(0.004)	(0.004)	(0.004)	(0.008)	(0.005)
Years in Congress	-0.021^{***}	-0.021^{***}	-0.022^{***}	-0.036^{***}	0.000
	(0.005)	(0.005)	(0.005)	(0.009)	(0.007)
Republican (vs. Democrat)	-0.447^{***}	-0.428^{***}	-0.382^{**}		
	(0.122)	(0.128)	(0.132)		
Senate (vs. House)	-0.811^{**}	-0.859^{**}			
	(0.277)	(0.272)			
Gender (Male)	0.013	0.026	0.041	−0.082	0.114
	(0.093)	(0.092)	(0.092)	(0.132)	(0.123)
Competitiveness	−0.011	−0.010	-0.013^{*}	−0.007	-0.029^{**}
	(0.006)	(0.006)	(0.006)	(0.009)	(0.010)
Ideological Extremity	-0.424^{***}	-0.418^{***}	-0.427^{***}	-0.714^{***}	-0.268^{*}
	(0.101)	(0.102)	(0.104)	(0.179)	(0.130)
Region Fixed Effects		✓			
State Fixed Effects			✓	✓	✓
R^2	0.141	0.146	0.313	0.378	0.461
Adj. R^2	0.127	0.126	0.215	0.223	0.325
Num. obs.	431	431	431	207	224

$^{***}p < 0.001$; $^{**}p < 0.01$; $^{*}p < 0.05$

Table S10: OLS: Regressing in-state donations (log) on critical debate. Robust standard errors in parenthesis.

	Pooled			Democrat	Republican
	Model 1	Model 2	Model 3	Model 4	Model 5
Personal attacks (%)	0.045	0.048	0.053	0.155^{*}	0.032
	(0.029)	(0.029)	(0.031)	(0.068)	(0.039)
Years in Congress	-0.011^{*}	-0.011^{*}	-0.011^{*}	-0.024^{**}	0.011
	(0.005)	(0.005)	(0.005)	(0.008)	(0.009)
Republican (vs. Democrat)	−0.136	−0.074	−0.158		
	(0.101)	(0.103)	(0.112)		
Senate (vs. House)	0.812	0.725			
	(0.446)	(0.452)			
Gender (Male)	−0.148	−0.155	−0.104	−0.148	−0.127
	(0.092)	(0.090)	(0.100)	(0.136)	(0.171)
Competitiveness	−0.011	−0.010	−0.012	−0.002	-0.023^{*}
	(0.006)	(0.006)	(0.006)	(0.011)	(0.010)
Ideological Extremity	-0.371^{***}	-0.358^{***}	-0.347^{**}	-0.620^{**}	−0.205
	(0.101)	(0.100)	(0.111)	(0.220)	(0.146)
Region Fixed Effects		✓			
State Fixed Effects			✓	✓	✓
R^2	0.111	0.124	0.227	0.349	0.246
Adj. R^2	0.096	0.103	0.116	0.186	0.055
Num. obs.	430	430	430	206	224

$^{***}p < 0.001$; $^{**}p < 0.01$; $^{*}p < 0.05$

Table S11: OLS: Regressing out-of-state donations on the relative personal attack score. Robust standard errors in parenthesis.

	Pooled			Democrat	Republican
	Model 1	Model 2	Model 3	Model 4	Model 5
Critical debate (%)	0.008	0.007	0.007	0.028^{**}	−0.001
	(0.005)	(0.005)	(0.005)	(0.010)	(0.007)
Years in Congress	-0.014^{**}	-0.013^{**}	-0.014^{*}	-0.032^{***}	0.010
	(0.005)	(0.005)	(0.006)	(0.009)	(0.009)
Republican (vs. Democrat)	−0.194	−0.114	−0.197		
	(0.125)	(0.130)	(0.143)		
Senate (vs. House)	0.797	0.713			
	(0.444)	(0.449)			
Gender (Male)	−0.148	−0.156	−0.112	−0.118	−0.150
	(0.092)	(0.091)	(0.101)	(0.136)	(0.177)
Competitiveness	−0.011	−0.010	−0.012	−0.005	-0.021^{*}
	(0.006)	(0.006)	(0.006)	(0.011)	(0.010)
Ideological Extremity	-0.410^{***}	-0.379^{**}	-0.374^{**}	-0.771^{**}	−0.128
	(0.121)	(0.122)	(0.137)	(0.237)	(0.182)
Region Fixed Effects		✓			
State Fixed Effects			✓	✓	✓
R^2	0.111	0.121	0.223	0.363	0.241
Adj. R^2	0.096	0.100	0.111	0.203	0.049
Num. obs.	430	430	430	206	224

$^{***}p < 0.001$; $^{**}p < 0.01$; $^{*}p < 0.05$

Table S12: OLS: Regressing out-of-state donations on the relative personal attack score. Robust standard errors in parenthesis.

	Pooled			Democrat	Republican
	Model 1	Model 2	Model 3	Model 4	Model 5
Personal attacks (%)	0.044	0.048	0.040	0.115*	0.032
	(0.025)	(0.025)	(0.027)	(0.053)	(0.038)
Years in Congress	−0.017***	−0.018***	−0.019***	−0.026**	−0.006
	(0.005)	(0.005)	(0.005)	(0.008)	(0.008)
Republican (vs. Democrat)	−0.326**	−0.258*	−0.159		
	(0.100)	(0.106)	(0.110)		
Senate (vs. House)	−0.081	−0.174			
	(0.303)	(0.299)			
Gender (Male)	−0.197*	−0.194*	−0.158	−0.186	−0.206
	(0.093)	(0.091)	(0.093)	(0.128)	(0.152)
Competitiveness	−0.023***	−0.022***	−0.021***	−0.019*	−0.021
	(0.006)	(0.005)	(0.006)	(0.009)	(0.011)
Ideological Extremity	−0.033	−0.026	−0.025	−0.081	0.015
	(0.088)	(0.088)	(0.090)	(0.158)	(0.136)
Region Fixed Effects		✓			
State Fixed Effects			✓	✓	✓
R^2	0.151	0.166	0.306	0.381	0.381
Adj. R^2	0.137	0.147	0.206	0.227	0.224
Num. obs.	431	431	431	207	224

$^{***}p < 0.001$; $^{**}p < 0.01$; $^{*}p < 0.05$

Table S13: OLS: Regressing number of in-state donors (log) on personal attacks. Robust standard errors in parenthesis.

	Pooled			Democrat	Republican
	Model 1	Model 2	Model 3	Model 4	Model 5
Critical debate (%)	0.011*	0.010*	0.009	0.020*	0.005
	(0.005)	(0.005)	(0.005)	(0.008)	(0.006)
Years in Congress	−0.021***	−0.021***	−0.022***	−0.032***	−0.008
	(0.005)	(0.005)	(0.005)	(0.009)	(0.008)
Republican (vs. Democrat)	−0.436***	−0.352**	−0.252		
	(0.118)	(0.124)	(0.130)		
Senate (vs. House)	−0.080	−0.167			
	(0.301)	(0.298)			
Gender (Male)	−0.199*	−0.195*	−0.167	−0.163	−0.234
	(0.093)	(0.091)	(0.093)	(0.131)	(0.151)
Competitiveness	−0.023***	−0.022***	−0.021***	−0.021*	−0.020
	(0.006)	(0.006)	(0.006)	(0.010)	(0.011)
Ideological Extremity	−0.110	−0.085	−0.090	−0.187	−0.002
	(0.104)	(0.104)	(0.108)	(0.164)	(0.153)
Region Fixed Effects		✓			
State Fixed Effects			✓	✓	✓
R^2	0.157	0.168	0.308	0.388	0.378
Adj. R^2	0.143	0.148	0.208	0.235	0.220
Num. obs.	431	431	431	207	224

$^{***}p < 0.001$; $^{**}p < 0.01$; $^{*}p < 0.05$

Table S14: OLS: Regressing number of in-state donors (log) on critical debate. Robust standard errors in parenthesis.

	Pooled			Democrat	Republican
	Model 1	Model 2	Model 3	Model 4	Model 5
Personal attacks (%)	0.057	0.060	0.056	0.133*	0.045
	(0.034)	(0.033)	(0.036)	(0.061)	(0.051)
Years in Congress	−0.014**	−0.014**	−0.014**	−0.021**	−0.004
	(0.005)	(0.005)	(0.005)	(0.007)	(0.010)
Republican (vs. Democrat)	0.090	0.141	0.085		
	(0.101)	(0.103)	(0.113)		
Senate (vs. House)	0.701	0.631			
	(0.461)	(0.465)			
Gender (Male)	−0.268**	−0.274**	−0.251*	−0.176	−0.373
	(0.090)	(0.089)	(0.100)	(0.119)	(0.203)
Competitiveness	−0.011*	−0.010	−0.011*	−0.006	−0.020
	(0.006)	(0.006)	(0.006)	(0.009)	(0.012)
Ideological Extremity	−0.149	−0.138	−0.146	−0.181	−0.145
	(0.103)	(0.102)	(0.111)	(0.183)	(0.176)
Region Fixed Effects		✓			
State Fixed Effects			✓	✓	✓
R^2	0.107	0.115	0.201	0.313	0.209
Adj. R^2	0.092	0.094	0.086	0.143	0.010
Num. obs.	432	432	432	207	225

$^{***}p < 0.001$; $^{**}p < 0.01$; $^{*}p < 0.05$

Table S15: OLS: Regressing number of in-state donors (log) on personal attacks. Robust standard errors in parenthesis.

	Pooled			Democrat	Republican
	Model 1	Model 2	Model 3	Model 4	Model 5
Critical debate (%)	0.004	0.003	0.004	0.016	0.001
	(0.005)	(0.005)	(0.006)	(0.009)	(0.008)
Years in Congress	-0.016^{***}	-0.015^{**}	-0.016^{**}	-0.026^{**}	-0.006
	(0.005)	(0.005)	(0.005)	(0.009)	(0.010)
Republican (vs. Democrat)	0.119	0.187	0.114		
	(0.126)	(0.129)	(0.142)		
Senate (vs. House)	0.651	0.583			
	(0.461)	(0.464)			
Gender (Male)	-0.265^{**}	-0.271^{**}	-0.255^{*}	-0.144	-0.406
	(0.091)	(0.091)	(0.101)	(0.120)	(0.207)
Competitiveness	-0.011^{*}	-0.011	-0.011^{*}	-0.007	-0.019
	(0.006)	(0.006)	(0.006)	(0.009)	(0.013)
Ideological Extremity	-0.124	-0.098	-0.125	-0.238	-0.086
	(0.122)	(0.121)	(0.136)	(0.198)	(0.211)
Region Fixed Effects		✓			
State Fixed Effects			✓	✓	✓
R^2	0.097	0.104	0.192	0.302	0.202
Adj. R^2	0.083	0.083	0.076	0.129	0.002
Num. obs.	432	432	432	207	225

$^{***}p < 0.001$; $^{**}p < 0.01$; $^{*}p < 0.05$

Table S16: OLS: Regressing number of in-state donors (log) on critical debate. Robust standard errors in parenthesis.

	Pooled			Democrat	Republican
	Model 1	Model 2	Model 3	Model 4	Model 5
Personal attacks (%)	−0.008	−0.008	−0.011	−0.014	−0.007
	(0.008)	(0.008)	(0.007)	(0.012)	(0.010)
Years in Congress	−0.001	−0.001	−0.001	0.000	−0.003
	(0.001)	(0.001)	(0.001)	(0.002)	(0.002)
Republican (vs. Democrat)	−0.041	−0.053	−0.008		
	(0.026)	(0.027)	(0.030)		
Senate (vs. House)	−0.374***	−0.365***			
	(0.059)	(0.061)			
Gender (Male)	0.054*	0.060**	0.052*	0.023	0.081*
	(0.022)	(0.022)	(0.023)	(0.032)	(0.035)
Competitiveness	0.001	0.001	0.001	0.000	−0.000
	(0.001)	(0.001)	(0.002)	(0.002)	(0.003)
Ideological Extremity	0.030	0.026	0.026	0.032	0.012
	(0.026)	(0.026)	(0.025)	(0.048)	(0.036)
Region Fixed Effects		✓			
State Fixed Effects			✓	✓	✓
R^2	0.096	0.119	0.255	0.320	0.327
Adj. R^2	0.081	0.098	0.148	0.152	0.157
Num. obs.	431	431	431	207	224

$^{***}p < 0.001$; $^{**}p < 0.01$; $^{*}p < 0.05$

Table S17: OLS: Regressing the ratio of in-state to out-of-state donations on personal attacks. Robust standard errors in parenthesis.

	Pooled			Democrat	Republican
	Model 1	Model 2	Model 3	Model 4	Model 5
Critical debate (%)	0.000	0.001	0.001	−0.001	0.002
	(0.001)	(0.001)	(0.001)	(0.002)	(0.002)
Years in Congress	−0.001	−0.001	−0.001	0.001	−0.003
	(0.001)	(0.001)	(0.001)	(0.002)	(0.002)
Republican (vs. Democrat)	-0.062^{*}	-0.080^{*}	−0.042		
	(0.031)	(0.032)	(0.036)		
Senate (vs. House)	-0.362^{***}	-0.352^{***}			
	(0.060)	(0.062)			
Gender (Male)	0.053^{*}	0.059^{**}	0.052^{*}	0.018	0.086^{*}
	(0.022)	(0.022)	(0.023)	(0.032)	(0.036)
Competitiveness	0.001	0.001	0.001	0.000	−0.001
	(0.001)	(0.001)	(0.002)	(0.002)	(0.003)
Ideological Extremity	0.014	0.006	0.002	0.029	−0.025
	(0.030)	(0.030)	(0.030)	(0.053)	(0.043)
Region Fixed Effects		✓			
State Fixed Effects			✓	✓	✓
R^2	0.093	0.117	0.250	0.316	0.327
Adj. R^2	0.078	0.096	0.142	0.146	0.157
Num. obs.	431	431	431	207	224

$^{***}p < 0.001$; $^{**}p < 0.01$; $^{*}p < 0.05$

Table S18: OLS: Regressing the ratio of in-state to out-of-state donations on critical debate. Robust standard errors in parenthesis.

	Pooled			Democrat	Republican
	Model 1	Model 2	Model 3	Model 4	Model 5
Personal attacks (%)	-0.006	-0.006	-0.008	-0.015	-0.006
	(0.008)	(0.008)	(0.008)	(0.012)	(0.010)
Years in Congress	0.000	0.000	-0.001	0.001	-0.001
	(0.001)	(0.001)	(0.001)	(0.002)	(0.002)
Republican (vs. Democrat)	-0.141^{***}	-0.139^{***}	-0.084^{**}		
	(0.027)	(0.028)	(0.030)		
Senate (vs. House)	-0.220^{**}	-0.224^{**}			
	(0.076)	(0.078)			
Gender (Male)	0.048^{*}	0.051^{*}	0.050^{*}	0.002	0.095^{*}
	(0.022)	(0.022)	(0.025)	(0.031)	(0.045)
Competitiveness	-0.003	-0.002	-0.001	-0.003	0.003
	(0.001)	(0.001)	(0.002)	(0.002)	(0.003)
Ideological Extremity	0.043	0.041	0.044	0.034	0.053
	(0.027)	(0.027)	(0.028)	(0.047)	(0.039)
Region Fixed Effects		✓			
State Fixed Effects			✓	✓	✓
R^2	0.143	0.153	0.264	0.238	0.267
Adj. R^2	0.128	0.133	0.159	0.048	0.083
Num. obs.	432	432	432	207	225

$^{***}p < 0.001$; $^{**}p < 0.01$; $^{*}p < 0.05$

Table S19: OLS: Regressing the ratio of in-state to out-of-state donors on personal attacks. Robust standard errors in parenthesis.

	Pooled			Democrat	Republican
	Model 1	Model 2	Model 3	Model 4	Model 5
Critical debate (%)	0.002	0.002	0.001	0.000	0.001
	(0.001)	(0.001)	(0.001)	(0.002)	(0.002)
Years in Congress	−0.000	−0.000	−0.001	0.001	−0.001
	(0.001)	(0.001)	(0.001)	(0.002)	(0.002)
Republican (vs. Democrat)	-0.181^{***}	-0.180^{***}	-0.121^{**}		
	(0.032)	(0.033)	(0.037)		
Senate (vs. House)	-0.203^{**}	-0.207^{**}			
	(0.075)	(0.078)			
Gender (Male)	0.046^{*}	0.050^{*}	0.049^{*}	−0.002	0.099^{*}
	(0.023)	(0.022)	(0.025)	(0.031)	(0.046)
Competitiveness	−0.003	−0.002	−0.001	−0.003	0.002
	(0.001)	(0.001)	(0.002)	(0.002)	(0.003)
Ideological Extremity	0.013	0.012	0.019	0.023	0.032
	(0.031)	(0.031)	(0.033)	(0.053)	(0.050)
Region Fixed Effects		✓			
State Fixed Effects			✓	✓	✓
R^2	0.145	0.155	0.264	0.232	0.265
Adj. R^2	0.130	0.135	0.158	0.041	0.081
Num. obs.	432	432	432	207	225

$^{***}p < 0.001$; $^{**}p < 0.01$; $^{*}p < 0.05$

Table S20: OLS: Regressing the ratio of in-state to out-of-state donors on critical debate. Robust standard errors in parenthesis.

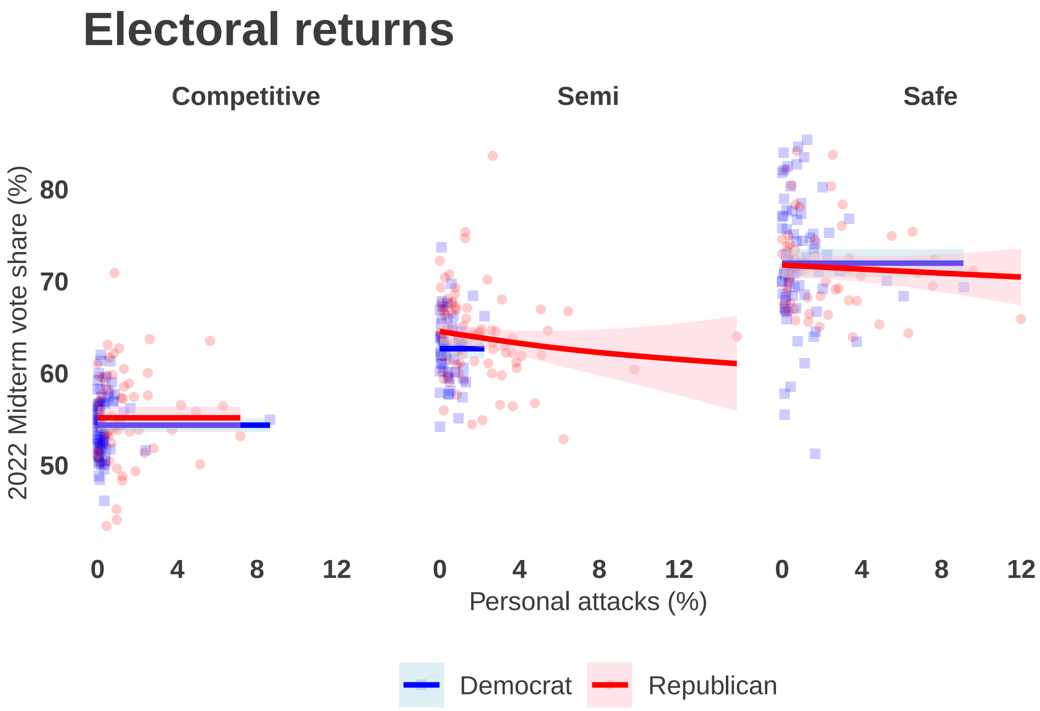

Figure S5: The relationship between personal attacks and election returns by district competitiveness (Cook PVI dataset).

	Pooled			Democrat	Republican
	Model 1	Model 2	Model 3	Model 4	Model 5
Personal attacks (%)	0.137***	0.136***	0.123***	0.208*	0.099**
	(0.031)	(0.031)	(0.030)	(0.081)	(0.033)
Years in Congress	0.008	0.007	0.007	−0.005	0.025**
	(0.005)	(0.005)	(0.006)	(0.008)	(0.008)
Republican (vs. Democrat)	−0.063	−0.059	−0.094		
	(0.114)	(0.122)	(0.131)		
Senate (vs. House)	0.669**	0.683**			
	(0.209)	(0.216)			
Gender (Male)	−0.033	−0.037	−0.056	−0.029	−0.125
	(0.097)	(0.097)	(0.102)	(0.153)	(0.148)
Competitiveness	0.004	0.004	0.003	0.022*	−0.022
	(0.006)	(0.006)	(0.007)	(0.010)	(0.012)
Ideological Extremity	0.146	0.145	0.162	−0.103	0.421**
	(0.113)	(0.114)	(0.115)	(0.208)	(0.156)
Region Fixed Effects		✓			
State Fixed Effects			✓	✓	✓
R^2	0.108	0.109	0.211	0.269	0.313
Adj. R^2	0.093	0.088	0.098	0.089	0.142
Num. obs.	440	440	440	213	227

$^{***}p < 0.001$; $^{**}p < 0.01$; $^{*}p < 0.05$

Table S21: OLS: Regressing media mentions (log and standardized) on personal attacks. Robust standard errors in parenthesis.

4.3 Media mentions

Table S21 shows linear models regressing media mentions on personal attacks. Table S22 shows a similar linear model regressing media mentions on critical debate.

	Pooled			Democrat	Republican
	Model 1	Model 2	Model 3	Model 4	Model 5
Critical debate (%)	0.020***	0.021***	0.024***	0.029**	0.020**
	(0.005)	(0.005)	(0.006)	(0.009)	(0.007)
Years in Congress	0.001	0.001	−0.001	−0.013	0.017*
	(0.006)	(0.006)	(0.006)	(0.009)	(0.009)
Republican (vs. Democrat)	−0.161	−0.196	−0.303*		
	(0.131)	(0.143)	(0.150)		
Senate (vs. House)	0.585**	0.640**			
	(0.214)	(0.219)			
Gender (Male)	−0.030	−0.036	−0.074	0.029	−0.213
	(0.099)	(0.100)	(0.103)	(0.153)	(0.150)
Competitiveness	0.004	0.004	0.002	0.021*	−0.022
	(0.006)	(0.006)	(0.006)	(0.010)	(0.012)
Ideological Extremity	0.080	0.065	0.017	−0.242	0.274
	(0.124)	(0.126)	(0.132)	(0.225)	(0.185)
Region Fixed Effects		✓			
State Fixed Effects			✓	✓	✓
R^2	0.089	0.092	0.213	0.270	0.308
Adj. R^2	0.074	0.071	0.100	0.090	0.136
Num. obs.	440	440	440	213	227

$^{***}p < 0.001$; $^{**}p < 0.01$; $^{*}p < 0.05$

Table S22: OLS: Regressing media mentions (log and standardized) on critical debate. Robust standard errors in parenthesis.

	Sponsor bill			Co-sponsor bill		
	Pooled	Democrat	Republican	Pooled	Democrat	Republican
Personal attacks (%)	1.480	−0.241	1.868	12.266	14.842	10.262
	(1.235)	(0.636)	(1.584)	(6.739)	(13.570)	(7.958)
Years in Congress	0.099	0.109	0.147	4.384***	5.142**	3.141*
	(0.066)	(0.076)	(0.132)	(1.120)	(1.660)	(1.340)
Republican (vs. Democrat)	0.001			−47.975**		
	(1.527)			(15.255)		
Independent (vs. Democrat)	−9.120***			−45.985		
	(2.656)			(25.946)		
Senate (vs. House)	18.931***	25.241***	12.716**	−1.780	17.240	−18.118
	(2.728)	(2.932)	(4.492)	(16.670)	(26.547)	(19.924)
R^2	0.068	0.418	0.025	0.053	0.074	0.019
Adj. R^2	0.060	0.411	0.014	0.044	0.063	0.007
Num. obs.	530	258	269	530	258	269

$^{***}p < 0.001$; $^{**}p < 0.01$; $^{*}p < 0.05$

Table S23: OLS: Regression (co-)sponsorship of bills on personal attacks. Robust standard errors reported.

4.4 Legislative effectiveness

Table S23 shows linear models regressing bill (co-)sponsorship in personal attacks. S24 shows a similar model with critical debate as a predictor.

	Sponsor bill			Co-sponsor bill		
	Pooled	Democrat	Republican	Pooled	Democrat	Republican
Critical debate (%)	0.127*	0.082	0.127	1.839	3.353*	1.002
	(0.054)	(0.078)	(0.095)	(0.997)	(1.637)	(1.211)
Years in Congress	0.117	0.044	0.424	3.949**	3.404	4.248*
	(0.083)	(0.074)	(0.296)	(1.320)	(1.776)	(2.100)
Republican (vs. Democrat)	6.326			−6.646		
	(6.268)			(36.508)		
Independent (vs. Democrat)	0.191			39.081		
	(3.149)			(29.557)		
Senate (vs. House)	6.367	11.590***	3.006	−65.052*	−54.359	−44.563
	(4.404)	(3.453)	(7.382)	(27.250)	(41.446)	(43.155)
Gender (Male)	0.536	−1.047	−0.285	−51.037*	−60.149*	−17.334
	(2.242)	(1.297)	(2.955)	(22.458)	(28.373)	(25.198)
Competitiveness	−0.325	0.155	−0.848	0.616	2.283	−3.350
	(0.314)	(0.100)	(0.556)	(1.953)	(2.014)	(3.043)
Ideological Extremity	8.573	−1.459	14.369	52.013	50.210	54.234
	(8.965)	(1.702)	(14.223)	(45.489)	(33.951)	(70.668)
R^2	0.033	0.058	0.046	0.091	0.192	0.032
Adj. R^2	0.014	0.030	0.020	0.074	0.168	0.006
Num. obs.	437	211	225	437	211	225

$^{***}p < 0.001$; $^{**}p < 0.01$; $^{*}p < 0.05$

Table S24: OLS: Regression (co-)sponsorship of bills on critical debate. Robust standard errors reported.

	Pooled			Democrat	Republican
	Model 1	Model 2	Model 3	Model 4	Model 5
Personal Attacks	488.464	447.434	315.455	3210.343	−242.287
	(401.569)	(411.636)	(488.586)	(3658.612)	(482.517)
Years Serving Congress	−1.454	−1.396	−0.657	−2.404	2.430
	(3.124)	(3.113)	(2.600)	(3.839)	(1.616)
Republican (vs. Democrat)	0.937	10.636	15.954		
	(19.322)	(16.935)	(27.020)		
Senate (vs. House)	−34.588	−36.014			
	(25.817)	(27.369)			
Gender (Male)	−2.335	−4.727	−13.638	10.510	−35.179
	(18.514)	(17.875)	(19.744)	(16.104)	(47.878)
Competitiveness	−1.387	−1.406	−3.233	−4.765	−3.511
	(0.863)	(0.910)	(1.923)	(3.189)	(2.663)
Ideological Extremity	10.602	13.288	21.414	52.471	14.826
	(15.610)	(16.247)	(21.242)	(34.254)	(27.726)
Region Fixed Effects		✓			
State Fixed Effects			✓	✓	✓
R^2	0.013	0.018	0.173	0.307	0.151
Adj. R^2	−0.012	−0.017	−0.023	0.042	−0.216
Num. obs.	289	289	289	142	147

$^{***}p < 0.001$; $^{**}p < 0.01$; $^{*}p < 0.05$

Table S25: OLS: Regressing percent change in net worth 2022 v. 2023 on personal attacks. Robust standard errors in parenthesis.

4.5 Net Worth

Table S25 shows linear models regressing networth in personal attacks. S26 shows a similar model with critical debate as a predictor.

	Pooled			Democrat	Republican
	Model 1	Model 2	Model 3	Model 4	Model 5
Critical Debate	−120.862	−142.050	−95.310	−115.046	−40.404
	(100.311)	(117.355)	(110.490)	(183.708)	(151.170)
Years Serving Congress	−1.667	−1.655	−0.843	−2.336	2.240
	(3.242)	(3.278)	(2.729)	(4.121)	(1.664)
Republican (vs. Democrat)	31.121	44.915	39.706		
	(22.701)	(27.392)	(27.417)		
Senate (vs. House)	−46.203	−47.661			
	(28.951)	(31.286)			
Gender (Male)	−0.725	−3.610	−13.102	23.896	−33.949
	(18.548)	(17.916)	(19.556)	(21.333)	(48.123)
Competitiveness	−1.382	−1.408	−3.217	−4.801	−3.583
	(0.859)	(0.907)	(1.903)	(3.141)	(2.672)
Ideological Extremity	32.734	37.870	38.195	93.407	16.223
	(22.952)	(25.901)	(26.503)	(47.403)	(34.642)
Region Fixed Effects		✓			
State Fixed Effects			✓	✓	✓
R^2	0.014	0.021	0.174	0.295	0.150
Adj. R^2	−0.011	−0.014	−0.021	0.025	−0.217
Num. obs.	289	289	289	142	147

$^{***}p < 0.001$; $^{**}p < 0.01$; $^{*}p < 0.05$

Table S26: OLS: Regressing percent change in net worth 2022 v. 2023 on critical debate. Robust standard errors in parenthesis.

	Pooled			Democrat	Republican
	Model 1	Model 2	Model 3	Model 4	Model 5
Personal Attacks	6.894	4.608	2.716	16.668	−0.419
	(7.317)	(7.327)	(7.453)	(21.627)	(7.734)
Years in Congress	−0.008	−0.010	−0.004	−0.018	0.032
	(0.018)	(0.019)	(0.019)	(0.029)	(0.024)
Republican (vs. Democrat)	-1.566^{***}	-1.762^{***}	-1.956^{***}		
	(0.337)	(0.356)	(0.374)		
Gender (Male)	0.254	0.215	0.043	0.336	−0.755
	(0.335)	(0.329)	(0.325)	(0.463)	(0.472)
Competitiveness	0.116^{***}	0.113^{***}	0.089^{***}	0.152^{***}	-0.065^{*}
	(0.023)	(0.023)	(0.023)	(0.032)	(0.028)
Ideological Extremity	-0.579^{*}	-0.620^{*}	−0.490	−1.008	0.251
	(0.287)	(0.290)	(0.300)	(0.532)	(0.357)
Region Fixed Effects		✓			
State Fixed Effects			✓	✓	✓
R^2	0.147	0.172	0.307	0.422	0.263
Adj. R^2	0.135	0.154	0.217	0.285	0.092
Num. obs.	429	429	429	210	219

$^{***}p < 0.001$; $^{**}p < 0.01$; $^{*}p < 0.05$

Table S27: OLS: Regressing district affective polarization on personal attacks (House only). Robust standard errors in parenthesis.

4.6 Constituent Preference

Table S27 shows linear models regressing bill (co-)sponsorship in personal attacks. S28 shows a similar model with critical debate as a predictor.

	Pooled			Democrat	Republican
	Model 1	Model 2	Model 3	Model 4	Model 5
Critical Debate	-3.623^{*}	-3.160^{*}	-1.806	-1.335	-1.157
	(1.519)	(1.506)	(1.428)	(2.617)	(1.619)
Years in Congress	-0.001	-0.004	-0.000	-0.014	0.034
	(0.018)	(0.018)	(0.019)	(0.029)	(0.023)
Republican (vs. Democrat)	-0.830^{*}	-1.112^{**}	-1.579^{***}		
	(0.398)	(0.411)	(0.443)		
Gender (Male)	0.276	0.229	0.057	0.388	-0.739
	(0.335)	(0.330)	(0.325)	(0.469)	(0.466)
Competitiveness	0.116^{***}	0.113^{***}	0.090^{***}	0.154^{***}	-0.063^{*}
	(0.023)	(0.023)	(0.023)	(0.032)	(0.028)
Ideological Extremity	-0.039	-0.163	-0.225	-0.782	0.427
	(0.349)	(0.348)	(0.355)	(0.624)	(0.427)
Region Fixed Effects		✓			
State Fixed Effects			✓	✓	✓
R^2	0.157	0.180	0.309	0.420	0.265
Adj. R^2	0.145	0.162	0.220	0.283	0.094
Num. obs.	429	429	429	210	219

$^{***}p < 0.001$; $^{**}p < 0.01$; $^{*}p < 0.05$

Table S28: OLS: Regressing district affective polarization on critical debate (House only). Robust standard errors in parenthesis.

5 Committee assignment

We run a series of linear probability models that estimate the relationship between personal attacks and assignments to committees and ranks. We identified whether a member of Congress was assigned to an important primary committee or not. To this end, we first classify the committee as either primary or secondary. The coding of committees is shown in Table S29.

Committee	Importance
Commission on Security and Cooperation in Europe	0
House Committee on Agriculture	0
House Committee on Appropriations	1
House Committee on Armed Services	1
House Committee on Education and the Workforce	0
House Committee on Energy and Commerce	0
House Committee on Ethics	0
House Committee on Financial Services	0
House Committee on Foreign Affairs	0
House Committee on Homeland Security	0
House Committee on House Administration	0
House Committee on Natural Resources	0
House Committee on Oversight and Accountability	0
House Committee on Rules	1
House Committee on Science, Space, and Technology	0
House Committee on Small Business	0
House Committee on the Budget	1
House Committee on the Judiciary	0
House Committee on Transportation and Infrastructure	1
House Committee on Veterans' Affairs	0
House Committee on Ways and Means	0
House Permanent Select Committee on Intelligence	0
House Select Committee US/Chinese Communist Party	0
House Select Subcommittee on the Coronavirus Pandemic	0
House Select Subcommittee on the Weaponization of Fed. Government	0
Joint Committee on Printing	0
Joint Committee on Taxation	0
Joint Committee on the Library	0
Joint Economic Committee	0
Senate Committee on Agriculture, Nutrition, and Forestry	0
Senate Committee on Appropriations	1
Senate Committee on Armed Services	1
Senate Committee on Banking, Housing, and Urban Affairs	0
Senate Committee on Commerce, Science, and Transportation	0
Senate Committee on Energy and Natural Resources	0

Senate Committee on Environment and Public Works	0
Senate Committee on Finance	0
Senate Committee on Foreign Relations	1
Senate Committee on Health, Education, Labor, and Pensions	0
Senate Committee on Homeland Security and Governmental Affairs	0
Senate Committee on Indian Affairs	0
Senate Committee on Rules and Administration	1
Senate Committee on Small Business and Entrepreneurship	0
Senate Committee on the Budget	1
Senate Committee on the Judiciary	0
Senate Committee on Veterans' Affairs	0
Senate Select Committee on Ethics	0
Senate Select Committee on Intelligence	0
Senate Special Committee on Aging	0
United States Senate Caucus on International Narcotics Control	0

Table S29: Committees in Congress and importance coding.

We then run linear probability models taking the following form:

$$y_{i,j} = \beta_0 + \beta_1 x_i + \mathbf{X}\boldsymbol{\beta} + \epsilon_{i,j}, \quad (2)$$

where y is legislator i's assignment to committee j, which can either take 1 (assigned to a primary committee) or 0 (assigned to a secondary committee), $\beta_1 x_i$ a parameter for legislator i's personal attack score, $\mathbf{X}\boldsymbol{\beta}$ a vector of parameters of control variables. Note that most members of Congress are assigned to several committees, which is why we consider each assignment separately. We cluster standard errors at the legislator level.

We repeat a similar analysis for the assignment of committee rank, where we define $y_{i,j}$ in Equation 2 as legislator i's assignment to the chair or ranking member of committee j (1) or as an ordinary member (0).

Table S30 shows the results of the linear probability model predicting committee assignments with personal attacks. Table S31 shows a similar model with critical debate as a predictor.

Table S32 reports the results for a similar analysis with the assignment of committee rank defined as the outcome variable and personal attacks as a predictor variable. Table S33 reports a similar regression variable with critical debate as a predictor variable.

	Pooled	Democrat		Republican	
	Model 1	House	Senate	House	Senate
Personal attacks (%)	−0.002	−0.006	0.021	−0.003	−0.024*
	(0.003)	(0.008)	(0.033)	(0.003)	(0.010)
Years in Congress	0.009***	0.009***	0.013***	0.010***	0.008*
	(0.001)	(0.002)	(0.002)	(0.002)	(0.002)
Republican (vs. Democrat)	0.019				
	(0.018)				
Senate (vs. House)	−0.006				
	(0.033)				
Gender (Male)	0.016	0.042	0.037	−0.016	−0.034
	(0.015)	(0.023)	(0.034)	(0.025)	(0.043)
Competitiveness	0.001	−0.001		0.003	
	(0.001)	(0.001)		(0.002)	
Ideological Extremity	−0.015	0.028	−0.110*	−0.020	0.041
	(0.014)	(0.026)	(0.046)	(0.017)	(0.026)
R^2	0.099	0.123	0.105	0.091	0.057
Adj. R^2	0.091	0.112	0.089	0.082	0.038
Num. obs.	938	399	219	491	206
N Clusters	431	205	48	214	49

$^{***}p < 0.001$; $^{**}p < 0.01$; $^{*}p < 0.05$

Table S30: OLS: Regressing committee rank (chair/ranking member vs. ordinary member) on personal attacks. Robust standard errors in parenthesis.

	Pooled	Democrat		Republican	
	Model 1	House	Senate	House	Senate
Critical debate (%)	0.001	0.001	−0.001	0.000	−0.002
	(0.001)	(0.001)	(0.002)	(0.001)	(0.021)
Years in Congress	0.009***	0.008***	0.013***	0.010***	0.014
	(0.001)	(0.002)	(0.002)	(0.003)	(0.014)
Republican (vs. Democrat)	0.005				
	(0.023)				
Senate (vs. House)	−0.001				
	(0.032)				
Gender (Male)	0.016	0.041	0.041	−0.012	−0.067
	(0.015)	(0.022)	(0.034)	(0.026)	(0.112)
Competitiveness	0.001	−0.001		0.003	0.008
	(0.001)	(0.001)		(0.002)	(0.016)
Ideological Extremity	−0.025	0.013	−0.074	−0.028	−0.005
	(0.018)	(0.029)	(0.041)	(0.025)	(0.368)
R^2	0.100	0.124	0.105	0.091	0.141
Adj. R^2	0.092	0.113	0.088	0.081	−0.038
Num. obs.	938	399	219	491	30
N Clusters	431	205	48	214	8

$^{***}p < 0.001$; $^{**}p < 0.01$; $^{*}p < 0.05$

Table S31: OLS: Regressing committee rank (chair/ranking member vs. ordinary member) on critical debate. Robust standard errors in parenthesis.

	Pooled	Democrat		Republican	
	Model 1	House	Senate	House	Senate
Personal attacks (%)	−0.008	−0.040*	−0.010	−0.002	0.088
	(0.007)	(0.013)	(0.048)	(0.009)	(0.184)
Years in Congress	0.004*	0.005*	0.004	0.004	−0.004
	(0.002)	(0.002)	(0.004)	(0.004)	(0.015)
Republican (vs. Democrat)	−0.013				
	(0.033)				
Senate (vs. House)	−0.067				
	(0.041)				
Gender (Male)	0.053	0.037	0.070	0.076	0.125
	(0.030)	(0.043)	(0.062)	(0.051)	(0.156)
Competitiveness	−0.002	−0.002		0.001	−0.001
	(0.002)	(0.003)		(0.003)	(0.014)
Ideological Extremity	−0.037	−0.053	0.030	−0.039	−0.137
	(0.030)	(0.050)	(0.111)	(0.041)	(0.197)
R^2	0.016	0.033	0.011	0.013	0.025
Adj. R^2	0.007	0.021	−0.008	0.002	−0.178
Num. obs.	938	399	219	491	30
N Clusters	431	205	48	214	8

$^{***}p < 0.001$; $^{**}p < 0.01$; $^{*}p < 0.05$

Table S32: OLS: Regressing committee importance on personal attacks. Robust standard errors in parenthesis.

	Pooled	Democrat		Republican	
	Model 1	House	Senate	House	Senate
Critical debate (%)	−0.000	−0.001	0.003	−0.001	−0.016
	(0.001)	(0.002)	(0.004)	(0.002)	(0.017)
Years in Congress	0.004*	0.005*	0.003	0.004	0.009
	(0.002)	(0.002)	(0.004)	(0.004)	(0.013)
Republican (vs. Democrat)	−0.020				
	(0.036)				
Senate (vs. House)	−0.059				
	(0.040)				
Gender (Male)	0.053	0.024	0.063	0.077	0.071
	(0.030)	(0.043)	(0.060)	(0.049)	(0.134)
Competitiveness	−0.002	−0.002		0.001	0.003
	(0.002)	(0.003)		(0.003)	(0.012)
Ideological Extremity	−0.042	−0.070	−0.022	−0.035	0.188
	(0.032)	(0.052)	(0.098)	(0.042)	(0.311)
R^2	0.015	0.024	0.014	0.013	0.037
Adj. R^2	0.006	0.012	−0.004	0.002	−0.163
Num. obs.	938	399	219	491	30
N Clusters	431	205	48	214	8

$^{***}p < 0.001$; $^{**}p < 0.01$; $^{*}p < 0.05$

Table S33: OLS: Regressing committee importance on critical debate. Robust standard errors in parenthesis.

6 Affect (MRP)

6.1 Multilevel Regression Model

We use a multilevel model to predict individual out-group affect scores. Each person's score is modeled as a function of a global intercept, random intercepts for their age group, gender, race, and congressional district, plus a fixed effect for the Trump 2020 two-party vote share in their district. The random intercepts allow each demographic and geographic group to deviate from the overall mean while sharing information across groups.

We use weakly informative priors: normal priors centered at zero for the intercept and Trump vote coefficient, and half-normal priors for all standard deviation parameters. The model is estimated using Hamiltonian Monte Carlo via `brms` with `cmdstanr`, running 4 chains of 2,000 iterations each (1,000 warmup, 1,000 sampling), yielding 4,000 posterior draws. Convergence is assessed using $\hat{R} < 1.01$, effective sample size > 400, and visual inspection of trace plots.

6.2 Synthetic Poststratification Frame

Poststratification requires the joint distribution of demographics and partisanship within districts, but the ACS provides only demographics and CCES samples are too small for reliable within-district crosstabs. We use `synthjoint` to generate synthetic joint distributions that simultaneously match ACS demographic margins exactly, approximate CCES district-level partisan shares, and respect the national demographic-partisanship relationship from our survey. This produces complete poststratification frames for all 435 congressional districts via constrained optimization.

6.3 Poststratification

For each posterior draw, we generate predictions for every cell in the poststratification frame and aggregate to the district level using population-weighted averages. This yields 4,000 plausible values per district. We report the posterior mean as our point estimate, the 95% credible interval for uncertainty, and the posterior standard deviation.
